# Supplementary material for: Turing’s children: Representation of sexual minorities in STEM
Source: PLoS One. 2020 Nov 18;15(11):e0241596. doi: 10.1371/journal.pone.0241596 (PMC7673532; doi:10.1371/journal.pone.0241596)
Supplement: S1 Text — (DOCX) [file pone.0241596.s001.docx]

**S1 Text. ACS variables description**

*Sex* reports whether an individual is male or female.

*In a same-sex couple*. The ACS does not directly ask individuals about their sexual orientation. However, the ACS identifies a primary reference person, defined as “the person living or staying here in whose name this house or apartment is owned, being bought, or rented”. The ACS also collects information on the relationship to the primary reference person for all members of the household, and the range of possible relationships includes husband, wife, and unmarried partner (as a different category than roommate or other nonrelative). By combining such information, we have created an indicator variable equal to one if an individual was in a same-sex couple; zero if an individual was in a different-sex couple. We have coded as individuals in a same-sex couple both married individuals and individuals living with an unmarried partner.

*Higher Education* is an indicator equal to one if an individual’s highest degree completed was a bachelor’s degree or higher (Master’s degree, Professional degree beyond a bachelor’s degree, Doctoral degree); zero otherwise.

*STEM degree* is an indicator equal to one if an individual received a bachelor’s degree in a STEM field; zero if they received their degree in a non-STEM field. This indicator refers to the primary field in which an individual received a bachelor’s degree. This indicator has been set to missing if an individual did not receive a bachelor’s degree. The following fields have been coded as STEM in line with the classification used by the U.S. Department of Commerce [Source: <https://files.eric.ed.gov/fulltext/ED594354.pdf> (Appendix Table 2)] and the U.S. Bureau of Labor Statistics [Source: <https://www.bls.gov/oes/topics.htm#stem>]:

- Agricultural Sciences
  - Animal Sciences
  - Food Science
  - Plant Science and Agronomy
  - Soil Science
- Environmental Science
- Architecture
- Communication Technologies
- Computer and Information Systems
  - Computer Programming and Data Processing
  - Computer Science
  - Information Sciences
  - Computer Information Management and Security
  - Computer Networking and Telecommunications
- General Engineering
  - Aerospace Engineering
  - Biological Engineering
  - Architectural Engineering
  - Biomedical Engineering
  - Chemical Engineering
  - Civil Engineering
  - Computer Engineering
  - Electrical Engineering
  - Engineering Mechanics, Physics, and Science
  - Environmental Engineering
  - Geological and Geophysical Engineering
  - Industrial and Manufacturing Engineering
  - Materials Engineering and Materials Science
  - Mechanical Engineering
  - Metallurgical Engineering
  - Mining and Mineral Engineering
  - Naval Architecture and Marine Engineering
  - Nuclear Engineering
  - Petroleum Engineering
  - Miscellaneous Engineering
- Engineering Technologies
  - Engineering and Industrial Management
  - Electrical Engineering Technology
  - Industrial Production Technologies
  - Mechanical Engineering Related Technologies
  - Miscellaneous Engineering Technologies
- Biology
  - Biochemical Sciences
  - Botany
  - Molecular Biology
  - Ecology
  - Genetics
  - Microbiology
  - Pharmacology
  - Physiology
  - Zoology
  - Neuroscience
  - Miscellaneous Biology
- Mathematics
  - Applied Mathematics
  - Statistics and Decision Science
- Military Technologies
- Interdisciplinary and Multi-Disciplinary Studies
  - Nutrition Sciences
  - Neuroscience
  - Mathematics and Computer Science
  - Cognitive Science and Biopsychology
- Physical Sciences
  - Astronomy and Astrophysics
  - Atmospheric Sciences and Meteorology
  - Chemistry
  - Geology and Earth Science
  - Geosciences
  - Oceanography
  - Physics
  - Materials Science
- Nuclear, Industrial Radiology, and Biological Technologies
- Transportation Sciences and Technologies
- Actuarial Science
- Operations, Logistics, and E-Commerce
- Management Information Systems and Statistics

*In the labor force* is an indicator equal to one if an individual was a part of the labor force, either working or seeking work, in the week preceding the interview; zero otherwise.

*Unemployed* is an indicator equal to one if an individual did not have a job, was looking for a job, and had not yet found one at the time of the interview, rather than being employed. Individuals not in the labor force have been coded as missing. Individuals who had never worked but were actively seeking their first job have been considered unemployed.

*STEM occupation* is an indicator equal to one if an individual’s primary occupation was in a STEM field; zero otherwise. This indicator has been set to missing if an individual was unemployed, with no work experience in the 5 years preceding the interview or earlier, or if they had never worked. Because of data limitations (i.e., no detailed codes for teachers), STEM postsecondary educators cannot be coded as STEM workers. In line with the classification used by the U.S. Department of Commerce [Source: <https://files.eric.ed.gov/fulltext/ED594354.pdf> (Appendix Table 1)] and the U.S. Bureau of Labor Statistics [Source: <https://www.bls.gov/oes/topics.htm#stem>], the following occupations have been coded as STEM:

- Management, Business, Science, and Arts Occupations
  - Computer and information systems managers
  - Architectural and Engineering Managers
  - Natural Sciences Managers
- Computer and Mathematical Occupations
  - Computer and Information Research Scientists
  - Computer Systems Analysts
  - Information security analysts
  - Computer Programmers
  - Software developers
  - Software quality assurance analysts and testers
  - Web Developers
  - Web and digital interface designers
  - Computer support specialists
  - Database Administrators
  - Network and Computer Systems Administrators
  - Computer Network Architects
  - Computer occupations, all other
  - Actuaries
  - Operations Research Analysts
  - Other mathematical science occupations
- Architecture and Engineering Occupations
  - Architects, Except landscape and Naval
  - Landscape architects
  - Surveyors, Cartographers, and Photogrammetrists
  - Aerospace Engineers
  - Biomedical and agricultural engineers
  - Chemical Engineers
  - Civil Engineers
  - Computer Hardware Engineers
  - Electrical and Electronics Engineers
  - Environmental Engineers
  - Industrial Engineers, including Health and Safety
  - Marine Engineers and Naval Architects
  - Materials Engineers
  - Mechanical Engineers
  - Petroleum, mining, and geological engineers, including mining safety engineers
  - Miscellaneous engineers including nuclear engineers
  - Drafters
  - Engineering Technicians, Except Drafters
  - Surveying and Mapping Technicians
- Life, Physical, and Social Science Occupations
  - Agricultural and Food Scientists
  - Biological Scientists
  - Conservation Scientists and Foresters
  - Other life scientists
  - Astronomers and Physicists
  - Atmospheric and Space Scientists
  - Chemists and Materials Scientists
  - Environmental scientists and specialists, including health
  - Geoscientists and hydrologists, except geographers
  - Physical Scientists, All Other
  - Agricultural and Food Science Technicians
  - Biological Technicians
  - Chemical Technicians
  - Environmental science and geoscience technicians, and nuclear technicians
  - Other life, physical, and social science technicians
  - Occupational health and safety specialists and technicians
- Sales Engineers

*Age* reports an individual’s age in years at the time of the interview.

*Race*. A series of indicator variables has been constructed to record an individual’s race: white, black or African American, Asian, or “other races”. Asian includes Chinese, Japanese, Other Asian or Pacific Islander. “Other races” include American Indian, Alaska Native, “other race not listed”, or individuals who selected two or three major races.

*Hispanic* is an indicator equal to one if an individual self-identified as Mexican, Puerto Rican, Cuban, or Other Hispanic; zero otherwise.

*Fertility* is an indicator equal to one if an individual had one or more own children (of any age or marital status) living in the household at the time of the interview, zero otherwise. This indicator includes step-children and adopted children as well as biological children. Similarly, another indicator has been constructed to be equal to one if an individual had one or more own children under age 5 living in the household at the time of the interview, zero otherwise.
