# Supplementary material for: Turing’s children: Representation of sexual minorities in STEM
Source: PLoS One. 2020 Nov 18;15(11):e0241596. doi: 10.1371/journal.pone.0241596 (PMC7673532; doi:10.1371/journal.pone.0241596)
Supplement: S2 Text — (DOCX) [file pone.0241596.s002.docx]

**S2 Text. NHIS variables description**

*Sex* reports whether an individual was male or female. The original NHIS variable is available for all individuals.

*Sexual orientation* reports an individual’s sexual orientation. The original NHIS variable is available for sample adults age 18 or older. The original question is the following:

Which of the following best represents how you think of yourself?

- Gay [lesbian or gay when asked to women]
- Straight, that is, not gay
- Bisexual
- Something else
- I don't know the answer
- Refused

*Higher Education* is an indicator equal to one if an individual’s highest degree completed was a bachelor’s degree or higher (Master’s degree, Professional degree beyond a bachelor’s degree, Doctoral degree); zero otherwise. This indicator has been set to missing if an individual refused to answer, did not know, or if the original NHIS variable is missing. The original NHIS variable is available for all individuals age 5 or older.

*In the labor force* is an indicator equal to one if an individual was a part of the labor force, either working (working for pay at job/business; working, without pay, at job/business; with job, but not at work) or seeking work (unemployed) in the week preceding the interview; zero otherwise. This indicator has been set to missing if an individual refused to answer, did not know, or if the original NHIS variable is missing. The original NHIS variable is available for all individuals age 18 or older.

*Unemployed* is an indicator equal to one if an individual did not have a job, was looking for a job, and had not yet found one at the time of the interview, rather than being employed. Individuals not in the labor force have been coded as missing. This indicator has been set to missing if an individual refused to answer, did not know, or if the original NHIS variable is missing. The original NHIS variable is available for all individuals age 18 or older.

*STEM occupation* is an indicator equal to one if an individual’s primary occupation was in a STEM field; zero otherwise. This indicator has been set to missing for individuals not in the universe, if an individual refused to answer, did not know, or if the original NHIS variable is missing. The original NHIS variable is available for sample adults age 18 or older who were working at a paying job in the week preceding the interview; with a job or business but not at work; working at a non-paying job in the week preceding the interview; or who had ever worked. The following occupations have been coded as STEM:

- Computer and Mathematical Occupations
  - Computer specialists
  - Mathematical science occupations
- Architecture and Engineering Occupations
  - Architects, surveyors, and cartographers
  - Engineers
  - Drafters, engineering, and mapping technicians
- Life, Physical, and Social Science Occupations
  - Life scientists
  - Physical scientists

*Age* reports an individual’s age in years at the time of the interview (top coded for 85 years or older). The original NHIS variable is available for all individuals.

*Race*. A series of indicator variables has been constructed to record an individual’s race: white, black or African American, Asian, or “other races”. Asian includes Chinese, Filipino, Asian Indian, or Other Asian. “Other races” include American Indian or Alaska Native, multiple races with no primary race selected, or individuals whose primary race was not releasable. The original NHIS variable is available for all individuals.

*Hispanic* is an indicator equal to one if an individual self-identified as Hispanic or Latino (Puerto Rican, Cuban or Cuban American, Dominican, Mexican or Mexican American, Central or South American, Other Latin American, Other Hispanic or Latino); zero otherwise. The original NHIS variable is available for all individuals.

*Fertility* is an indicator equal to one if an individual had one or more own children (of any age or marital status) living in the household at the time of the interview, zero otherwise. This indicator includes step-children and adopted children as well as biological children. The original NHIS variable is available for all individuals. Similarly, another indicator has been constructed to be equal to one if an individual had one or more own children under age 5 living in the household at the time of the interview, zero otherwise.
