# Supplementary material for: Turing’s children: Representation of sexual minorities in STEM
Source: PLoS One. 2020 Nov 18;15(11):e0241596. doi: 10.1371/journal.pone.0241596 (PMC7673532; doi:10.1371/journal.pone.0241596)
Supplement: S1 Fig — Panel A: STEM degrees. Panel B: STEM occupations. Notes: This map shows the gaps in STEM degrees and STEM occupations between men in same-sex couples and men in different-sex couples in each state. Darker colors indicate that the gaps between the share of men in same-sex couples and the share of men in different-sex couples working or with a STEM degree is smaller (or even positive). Weighed shares using person weights. See also Data and Methodology. Source: ACS 2009–2018. (DOCX) [file pone.0241596.s003.docx]

**S1 Fig.** **STEM degree and STEM occupation gaps between men in same-sex couples and men in different-sex couples, by state (ACS 2009-2018).**

**Panel A: STEM degrees.**

**Panel B: STEM occupations.**

Notes: This map shows the gaps in STEM degrees and STEM occupations between men in same-sex couples and men in different-sex couples in each state. Darker colors indicate that the gaps between the share of men in same-sex couples and the share of men in different-sex couples working or with a STEM degree is smaller (or even positive). Weighed shares using person weights. See also Data and Methodology. Source: ACS 2009-2018.
