# Supplementary material for: Turing’s children: Representation of sexual minorities in STEM
Source: PLoS One. 2020 Nov 18;15(11):e0241596. doi: 10.1371/journal.pone.0241596 (PMC7673532; doi:10.1371/journal.pone.0241596)
Supplement: S2 Fig — Panel A: STEM degrees. Panel B: STEM occupations. Notes: The vertical axis measures the share of women in same-sex couples over all coupled women in same-sex or different-sex couples in each field/occupation. Overall, 1.27% of women in a couple are in a same-sex couple. The horizontal axis measures the share of women (of any marital status and relation to the household head, age 18–65, sex not imputed) over all individuals in each field/occupation. Weighed shares using person weights. See also Data and Methodology. Only STEM fields/occupations reported. The dashed line plots the linear fit. Source: ACS 2009–2018. (DOCX) [file pone.0241596.s004.docx]

**S2 Fig. Relationship between share of coupled women in same-sex couples and share women in STEM degrees and STEM occupations (ACS 2009-2018).**

**Panel A: STEM degrees.**

**Panel B: STEM occupations.**

Notes: The vertical axis measures the share of women in same-sex couples over all coupled women in same-sex or different-sex couples in each field/occupation. Overall, 1.27% of women in a couple are in a same-sex couple. The horizontal axis measures the share of women (of any marital status and relation to the household head, age 18-65, sex not imputed) over all individuals in each field/occupation. Weighed shares using person weights. See also Data and Methodology. Only STEM fields/occupations reported. The dashed line plots the linear fit. Source: ACS 2009-2018.
