# Supplementary material for: Turing’s children: Representation of sexual minorities in STEM
Source: PLoS One. 2020 Nov 18;15(11):e0241596. doi: 10.1371/journal.pone.0241596 (PMC7673532; doi:10.1371/journal.pone.0241596)
Supplement: S3 Fig — Weight by field/occupation size. Panel A: STEM degrees. Panel B: STEM occupations. Notes: Compare to Fig 3. See also Data and Methodology. The vertical axis measures the share of men in same-sex couples over all coupled women in same-sex or different-sex couples in each field/occupation. Overall, 1.24% of men in a couple are in a same-sex couple. The horizontal axis measures the share of women (of any marital status and relation to the household head, age 18–65, sex not imputed) over all individuals in each field/occupation. Weighted shares using person weights. Only STEM fields/occupations reported. Each circle is proportional to the number of degree holders or workers (of any marital status and relation to the household head, age 18–65, men and women, sex not imputed) in that specific field/occupation. The dashed line plots the linear fit using field/occupation sizes as weights. Source: ACS 2009–2018. (DOCX) [file pone.0241596.s005.docx]

**S3 Fig. Relationship between share of coupled men in same-sex couples and share women in STEM degrees and STEM occupations (ACS 2009-2018). Weight by field/occupation size.**

**Panel A: STEM degrees.**

**Panel B: STEM occupations.**

Notes: Compare to Figure 3. See also Data and Methodology. The vertical axis measures the share of men in same-sex couples over all coupled women in same-sex or different-sex couples in each field/occupation. Overall, 1.24% of men in a couple are in a same-sex couple. The horizontal axis measures the share of women (of any marital status and relation to the household head, age 18-65, sex not imputed) over all individuals in each field/occupation. Weighted shares using person weights. Only STEM fields/occupations reported. Each circle is proportional to the number of degree holders or workers (of any marital status and relation to the household head, age 18-65, men and women, sex not imputed) in that specific field/occupation. The dashed line plots the linear fit using field/occupation sizes as weights. Source: ACS 2009-2018.
