# Supplementary material for: Turing’s children: Representation of sexual minorities in STEM
Source: PLoS One. 2020 Nov 18;15(11):e0241596. doi: 10.1371/journal.pone.0241596 (PMC7673532; doi:10.1371/journal.pone.0241596)
Supplement: S5 Fig — Panel A: STEM degrees. Panel B: STEM occupations. Notes: The vertical axis measures the share of men in same-sex couples over all coupled men in each field/occupation. Overall, 1.24% of men in a couple are in a same-sex couple. The horizontal axis measures the share of individuals (of any marital status and relation to the household head, age 18–65, men and women, sex not imputed) with any disability over all men in each field/occupation. Overall, 10.7% of individuals (of any marital status and relation to the household head, age 18–65, men and women, sex not imputed) have a disability coded in the IPUMS ACS as ambulatory difficulty, independent living difficulty, cognitive difficulty, self-care difficulty, vision or hearing difficulty. Weighed shares using person weights. See also Data and Methodology. Only STEM fields/occupations reported. The dashed line plots the linear fit. Source: ACS 2009–2018. (DOCX) [file pone.0241596.s007.docx]

**S5 Fig. Relationship between share of coupled men in same-sex couples and share of individuals with any disability in STEM degrees and STEM occupations (ACS 2009-2018).**

**Panel A: STEM degrees.**

**Panel B: STEM occupations.**

Notes: The vertical axis measures the share of men in same-sex couples over all coupled men in each field/occupation. Overall, 1.24% of men in a couple are in a same-sex couple. The horizontal axis measures the share of individuals (of any marital status and relation to the household head, age 18-65, men and women, sex not imputed) with any disability over all men in each field/occupation. Overall, 10.7% of individuals (of any marital status and relation to the household head, age 18-65, men and women, sex not imputed) have a disability coded in the IPUMS ACS as ambulatory difficulty, independent living difficulty, cognitive difficulty, self-care difficulty, vision or hearing difficulty. Weighed shares using person weights. See also Data and Methodology. Only STEM fields/occupations reported. The dashed line plots the linear fit. Source: ACS 2009-2018.
