# Supplementary material for: Turing’s children: Representation of sexual minorities in STEM
Source: PLoS One. 2020 Nov 18;15(11):e0241596. doi: 10.1371/journal.pone.0241596 (PMC7673532; doi:10.1371/journal.pone.0241596)
Supplement: S1 Table — ACS extensions. (DOCX) [file pone.0241596.s008.docx]

**S1 Table. STEM degrees and occupations by type of couple. ACS extensions.**

|  | Women | | |  | Men | | |
| --- | --- | --- | --- | --- | --- | --- | --- |
|  | Same-sex | Different-sex | Gap |  | Same-sex | Different-sex | Gap |
| *STEM degrees:* |  |  |  |  |  |  |  |
| As in Table 1 | 0.140 | 0.139 | 0.001 |  | 0.228 | 0.348 | -0.120^***^ |
| With STEM education | 0.144 | 0.143 | 0.001 |  | 0.230 | 0.351 | -0.121^***^ |
| Without biology | 0.091 | 0.093 | -0.002 |  | 0.172 | 0.297 | -0.125^***^ |
| Without food/nutrition | 0.137 | 0.135 | 0.002 |  | 0.226 | 0.347 | -0.121^***^ |
| With secondary field | 0.149 | 0.145 | 0.004 |  | 0.238 | 0.357 | -0.119^***^ |
| With health | 0.223 | 0.259 | -0.036^***^ |  | 0.276 | 0.375 | -0.099^***^ |
| *STEM occupations:* |  |  |  |  |  |  |  |
| As in Table 1 | 0.050 | 0.032 | 0.018^***^ |  | 0.084 | 0.095 | -0.011^***^ |
| Without agriculture/food | 0.049 | 0.032 | 0.017^***^ |  | 0.084 | 0.094 | -0.010^***^ |
| With physicians/surgeons | 0.051 | 0.033 | 0.018^***^ |  | 0.086 | 0.096 | -0.010^***^ |
| With health (all) | 0.177 | 0.179 | -0.002 |  | 0.164 | 0.131 | 0.033^***^ |
| Only with STEM degrees | 0.263 | 0.259 | 0.004 |  | 0.317 | 0.406 | -0.089^***^ |
| *Additional variables:* |  |  |  |  |  |  |  |
| In the labor force | 0.842 | 0.706 | 0.136^***^ |  | 0.852 | 0.884 | -0.032^***^ |
| Unemployed | 0.048 | 0.050 | -0.002^**^ |  | 0.049 | 0.044 | 0.005^***^ |
| Observations | 73,000 | 5,572,796 |  |  | 69,641 | 5,237,089 |  |

Notes: Weighed statistics using person weights. See also Data and Methodology, as well as Table 1. *STEM degree with education* counts as STEM also: Mathematics Teacher Education; Science and Computer Teacher Education. *STEM degree without biology* does not count as STEM: Biology; Biochemical Sciences; Botany; Molecular Biology; Ecology; Genetics; Microbiology; Pharmacology; Physiology; Zoology; Neuroscience; Miscellaneous Biology. *STEM degree without food/nutrition* does not count as STEM: Food Science; Nutrition Sciences. *STEM degree with secondary field* indicates whether an individual received a bachelor’s degree with a primary or secondary concentration in STEM. *STEM degree with health* includes as STEM all majors in the “General Medical and Health Services” IPUMS category. *STEM occupation without agriculture/food* does not count as STEM: agriculture and food scientists; agriculture and food science technicians. *STEM occupation with physicians/surgeons* counts as STEM occupations physicians and surgeons. *STEM occupation with health (all)* counts as STEM occupations all healthcare practitioners, healthcare technical occupations, and healthcare support occupations. *STEM occupation only with STEM degree* is the share of respondents with a STEM occupation only considering respondents with a STEM degree. *Unemployment rate* is computed only among the individuals in the labor force. “Observations” refers to the total number of respondents in the relevant sub-group. Source: ACS 2009-2018. ^*^ *p* < 0.10, ^**^ *p* < 0.05, ^***^ *p* < 0.01
