# Supplementary material for: Turing’s children: Representation of sexual minorities in STEM
Source: PLoS One. 2020 Nov 18;15(11):e0241596. doi: 10.1371/journal.pone.0241596 (PMC7673532; doi:10.1371/journal.pone.0241596)
Supplement: S2 Table — (DOCX) [file pone.0241596.s009.docx]

**S2 Table. STEM degrees and occupations by type of couple and by race.**

|  | In same-sex couples | | |  | In different-sex couples | | |
| --- | --- | --- | --- | --- | --- | --- | --- |
|  | White | Black | Asian |  | White | Black | Asian |
| *Women:* |  |  |  |  |  |  |  |
| STEM degrees | 0.136 | 0.125 | 0.289 |  | 0.120 | 0.115 | 0.301 |
| STEM occupations | 0.050 | 0.030 | 0.122 |  | 0.028 | 0.024 | 0.106 |
| Observations | 60,801 | 5,384 | 2,018 |  | 4,574,437 | 331,965 | 340,241 |
| *Men:* |  |  |  |  |  |  |  |
| STEM degrees | 0.216 | 0.206 | 0.388 |  | 0.321 | 0.275 | 0.613 |
| STEM occupations | 0.087 | 0.043 | 0.152 |  | 0.091 | 0.055 | 0.255 |
| Observations | 58,563 | 3,546 | 3,055 |  | 4,308,142 | 348,464 | 270,806 |

Notes: Weighed statistics using person weights. See also Data and Methodology, as well as Table 1. “Observations” refers to the total number of respondents in the relevant sub-group. Source: ACS 2009-2018. ^*^ *p* < 0.10, ^**^ *p* < 0.05, ^***^ *p* < 0.01
