# Supplementary material for: Turing’s children: Representation of sexual minorities in STEM
Source: PLoS One. 2020 Nov 18;15(11):e0241596. doi: 10.1371/journal.pone.0241596 (PMC7673532; doi:10.1371/journal.pone.0241596)
Supplement: S3 Table — (DOCX) [file pone.0241596.s010.docx]

**S3 Table. STEM degrees and occupations by type of couple and by age groups.**

|  | In same-sex couples | | |  | In different-sex couples | | |
| --- | --- | --- | --- | --- | --- | --- | --- |
|  | 18-34 | 35-49 | 50-65 |  | 18-34 | 35-49 | 50-65 |
| *Women:* |  |  |  |  |  |  |  |
| STEM degrees | 0.147 | 0.144 | 0.146 |  | 0.163 | 0.146 | 0.152 |
| STEM occupations | 0.042 | 0.055 | 0.049 |  | 0.034 | 0.035 | 0.035 |
| Observations | 19,659 | 25,864 | 47,559 |  | 1,204,997 | 1,996,685 | 3,358,213 |
| *Men:* |  |  |  |  |  |  |  |
| STEM degrees | 0.251 | 0.230 | 0.235 |  | 0.350 | 0.358 | 0.356 |
| STEM occupations | 0.080 | 0.092 | 0.087 |  | 0.096 | 0.103 | 0.100 |
| Observations | 15,528 | 26,533 | 44,309 |  | 966,979 | 1,902,722 | 3,019,751 |

Notes: Weighed statistics using person weights. See also Data and Methodology, as well as Table 1. “Observations” refers to the total number of respondents in the relevant sub-group. Source: ACS 2009-2018. ^*^ *p* < 0.10, ^**^ *p* < 0.05, ^***^ *p* < 0.01
