# Supplementary material for: Turing’s children: Representation of sexual minorities in STEM
Source: PLoS One. 2020 Nov 18;15(11):e0241596. doi: 10.1371/journal.pone.0241596 (PMC7673532; doi:10.1371/journal.pone.0241596)
Supplement: S4 Table — No controls. (DOCX) [file pone.0241596.s011.docx]

**S4 Table. STEM degree and STEM occupation gaps as in Table 3. No controls.**

|  | ACS 2009-2018 | | | |  | NHIS 2013-2018 | |
| --- | --- | --- | --- | --- | --- | --- | --- |
|  | STEM  degree | | STEM  occupation | |  | STEM  occupation | |
|  | Women | Men | Women | Men |  | Women | Men |
|  | (1) | (2) | (3) | (4) |  | (5) | (6) |
| In a same-sex couple | 0.001 | -0.120^***^ | 0.018^***^ | -0.010^***^ |  |  |  |
|  | (0.002) | (0.003) | (0.001) | (0.001) |  |  |  |
| Gay or lesbian |  |  |  |  |  | 0.004 | -0.023^***^ |
|  |  |  |  |  |  | (0.006) | (0.008) |
| Bisexual |  |  |  |  |  | 0.007 | -0.011 |
|  |  |  |  |  |  | (0.007) | (0.016) |
| Something else |  |  |  |  |  | -0.003 | -0.021 |
|  |  |  |  |  |  | (0.015) | (0.019) |
| Dependent variable mean | 0.139 | 0.345 | 0.032 | 0.095 |  | 0.030 | 0.087 |
| R-squared | 0.000 | 0.001 | 0.000 | 0.000 |  | 0.000 | 0.000 |
| Observations | 2,063,090 | 1,850,340 | 4,664,190 | 4,992,047 |  | 69,972 | 61,890 |

Notes: The dependent variable in columns 1-2 is whether an individual received a bachelor’s degree in a STEM field. The dependent variable in columns 3-6 is whether an individual used to work in a STEM occupation. Compare to Table 3. See also Data and Methodology. All variables are defined in detail in the SI. Weighted regressions using person weights. Standard errors in parentheses. Source: ACS 2009-2018 and NHIS 2013-2018. ^*^ *p* < 0.10, ^**^ *p* < 0.05, ^***^ *p* < 0.01
