# Supplementary material for: Turing’s children: Representation of sexual minorities in STEM
Source: PLoS One. 2020 Nov 18;15(11):e0241596. doi: 10.1371/journal.pone.0241596 (PMC7673532; doi:10.1371/journal.pone.0241596)
Supplement: S6 Table — Control for race/ethnicity. (DOCX) [file pone.0241596.s013.docx]

**S6 Table. STEM degree and STEM occupation gaps as in Table 3. Control for race/ethnicity.**

|  | ACS 2009-2018 | | | |  | NHIS 2013-2018 | |
| --- | --- | --- | --- | --- | --- | --- | --- |
|  | STEM  degree | | STEM  occupation | |  | STEM  occupation | |
|  | Women | Men | Women | Men |  | Women | Men |
|  | (1) | (2) | (3) | (4) |  | (5) | (6) |
| In a same-sex couple | 0.013^***^ | -0.111^***^ | 0.020^***^ | -0.010^***^ |  |  |  |
|  | (0.002) | (0.003) | (0.001) | (0.001) |  |  |  |
| Gay or lesbian |  |  |  |  |  | 0.006 | -0.018^**^ |
|  |  |  |  |  |  | (0.006) | (0.008) |
| Bisexual |  |  |  |  |  | 0.009 | -0.015 |
|  |  |  |  |  |  | (0.007) | (0.015) |
| Something else |  |  |  |  |  | -0.000 | -0.017 |
|  |  |  |  |  |  | (0.015) | (0.020) |
| Dependent variable mean | 0.139 | 0.345 | 0.032 | 0.095 |  | 0.030 | 0.087 |
| R-squared | 0.025 | 0.035 | 0.012 | 0.024 |  | 0.012 | 0.028 |
| Observations | 2,063,090 | 1,850,340 | 4,664,190 | 4,992,047 |  | 69,972 | 61,890 |

Notes: The dependent variable in columns 1-2 is whether an individual received a bachelor’s degree in a STEM field. The dependent variable in columns 3-6 is whether an individual used to work in a STEM occupation. Compare to Table 3. See also Data and Methodology. All variables are defined in detail in the SI. All regressions include controls for demographic characteristics (race and ethnicity only). Weighted regressions using person weights. Standard errors in parentheses. Source: ACS 2009-2018 and NHIS 2013-2018. ^*^ *p* < 0.10, ^**^ *p* < 0.05, ^***^ *p* < 0.01
