# Supplementary material for: Turing’s children: Representation of sexual minorities in STEM
Source: PLoS One. 2020 Nov 18;15(11):e0241596. doi: 10.1371/journal.pone.0241596 (PMC7673532; doi:10.1371/journal.pone.0241596)
Supplement: S10 Table — Add year fixed-effects. (DOCX) [file pone.0241596.s017.docx]

**S10 Table. STEM degree and STEM occupation gaps as in Table 3. Add year fixed-effects.**

|  | ACS 2009-2018 | | | |  | NHIS 2013-2018 | |
| --- | --- | --- | --- | --- | --- | --- | --- |
|  | STEM  degree | | STEM  occupation | |  | STEM  occupation | |
|  | Women | Men | Women | Men |  | Women | Men |
|  | (1) | (2) | (3) | (4) |  | (5) | (6) |
| In a same-sex couple | 0.010^***^ | -0.107^***^ | 0.017^***^ | -0.017^***^ |  |  |  |
|  | (0.002) | (0.003) | (0.001) | (0.001) |  |  |  |
| Gay or lesbian |  |  |  |  |  | 0.003 | -0.014^*^ |
|  |  |  |  |  |  | (0.006) | (0.008) |
| Bisexual |  |  |  |  |  | 0.006 | -0.016 |
|  |  |  |  |  |  | (0.007) | (0.015) |
| Something else |  |  |  |  |  | -0.004 | -0.017 |
|  |  |  |  |  |  | (0.014) | (0.020) |
| Dependent variable mean | 0.139 | 0.345 | 0.032 | 0.095 |  | 0.030 | 0.087 |
| R-squared | 0.031 | 0.039 | 0.016 | 0.030 |  | 0.013 | 0.030 |
| Observations | 2,063,090 | 1,850,340 | 4,664,190 | 4,992,047 |  | 69,972 | 61,890 |

Notes: The dependent variable in columns 1-2 is whether an individual received a bachelor’s degree in a STEM field. The dependent variable in columns 3-6 is whether an individual used to work in a STEM occupation. Compare to Table 3. See also Data and Methodology. All variables are defined in detail in the SI. All regressions include controls for demographic characteristics (age, race, ethnicity), fertility (indicators for children in the household and children under 5 in the household), location (state fixed effects in the ACS, region fixed effects in the NHIS since we do not observe state of residence in the NHIS public-use data), and year fixed effects. Weighted regressions using person weights. Standard errors in parentheses. Source: ACS 2009-2018 and NHIS 2013-2018. ^*^ *p* < 0.10, ^**^ *p* < 0.05, ^***^ *p* < 0.01
