# Supplementary material for: Turing’s children: Representation of sexual minorities in STEM
Source: PLoS One. 2020 Nov 18;15(11):e0241596. doi: 10.1371/journal.pone.0241596 (PMC7673532; doi:10.1371/journal.pone.0241596)
Supplement: S12 Table — (DOCX) [file pone.0241596.s019.docx]

**S12 Table. Figure 1 (STEM degrees) in tabular form.**

|  | #Degree  holders | Share of coupled men in same-sex couple | Share of individuals that is female |
| --- | --- | --- | --- |
| Biology | 192,073 | 0.019 | 0.524 |
| Computer Science | 115,480 | 0.013 | 0.267 |
| Electrical Engineering | 105,725 | 0.007 | 0.140 |
| Mechanical Engineering | 84,035 | 0.005 | 0.107 |
| Mathematics | 72,266 | 0.014 | 0.434 |
| Chemistry | 63,162 | 0.015 | 0.400 |
| General Engineering | 52,817 | 0.006 | 0.168 |
| Civil Engineering | 50,682 | 0.005 | 0.179 |
| Architecture | 41,906 | 0.036 | 0.343 |
| Computer and Information Systems | 37,226 | 0.012 | 0.322 |
| Chemical Engineering | 31,700 | 0.010 | 0.297 |
| Physics | 29,425 | 0.008 | 0.202 |
| Computer Engineering | 28,244 | 0.011 | 0.204 |
| Management Information Systems and Statistics | 22,339 | 0.014 | 0.367 |
| Biochemical Sciences | 21,525 | 0.024 | 0.453 |
| Industrial and Manufacturing Engineering | 20,868 | 0.009 | 0.263 |
| Geology and Earth Science | 18,914 | 0.010 | 0.308 |
| Environmental Science | 18,528 | 0.012 | 0.480 |
| Animal Sciences | 17,871 | 0.010 | 0.556 |
| Transportation Sciences and Technologies | 15,008 | 0.009 | 0.114 |
| Microbiology | 13,916 | 0.021 | 0.555 |
| Zoology | 13,468 | 0.015 | 0.435 |
| Information Sciences | 11,583 | 0.014 | 0.318 |
| Plant Science and Agronomy | 11,517 | 0.015 | 0.324 |
| Electrical Engineering Technology | 11,493 | 0.005 | 0.166 |
| Nutrition Sciences | 11,357 | 0.032 | 0.877 |
| Physiology | 11,198 | 0.017 | 0.544 |
| Aerospace Engineering | 11,171 | 0.007 | 0.113 |
| Industrial Production Technologies | 9,382 | 0.005 | 0.103 |
| Miscellaneous Engineering | 8,690 | 0.006 | 0.214 |
| Miscellaneous Engineering Technologies | 8,479 | 0.003 | 0.205 |
| Operations, Logistics and E-Commerce | 8,370 | 0.011 | 0.326 |
| Molecular Biology | 7,881 | 0.032 | 0.526 |
| Communication Technologies | 7,865 | 0.027 | 0.354 |
| Ecology | 7,761 | 0.018 | 0.556 |
| Miscellaneous Biology | 6,826 | 0.016 | 0.487 |
| Computer Networking and Telecommunications | 6,515 | 0.013 | 0.303 |
| Computer Information Management and Security | 6,297 | 0.014 | 0.264 |
| Biological Engineering | 5,503 | 0.008 | 0.277 |
| Biomedical Engineering | 5,349 | 0.017 | 0.393 |
| Statistics and Decision Science | 4,786 | 0.022 | 0.467 |
| Engineering Technologies | 4,758 | 0.005 | 0.218 |
| Engineering and Industrial Management | 4,724 | 0.004 | 0.198 |
| Applied Mathematics | 4,182 | 0.018 | 0.388 |
| Food Science | 4,154 | 0.017 | 0.614 |
| Materials Engineering and Materials Science | 3,892 | 0.008 | 0.277 |
| Neuroscience | 3,863 | 0.035 | 0.574 |
| Mechanical Engineering Related Technologies | 3,835 | 0.003 | 0.065 |
| Engineering Mechanics, Physics, and Science | 3,471 | 0.010 | 0.149 |
| Computer Programming and Data Processing | 3,412 | 0.017 | 0.338 |
| Environmental Engineering | 3,048 | 0.011 | 0.386 |
| Botany | 2,958 | 0.033 | 0.548 |
| Atmospheric Sciences and Meteorology | 2,612 | 0.021 | 0.263 |
| Petroleum Engineering | 2,487 | 0.009 | 0.152 |
| Materials Science | 2,348 | 0.010 | 0.287 |
| Architectural Engineering | 2,169 | 0.010 | 0.244 |
| *(cont.)* |  |  |  |
| Naval Architecture and Marine Engineering | 2,074 | 0.009 | 0.068 |
| Genetics | 2,002 | 0.019 | 0.557 |
| Oceanography | 1,970 | 0.008 | 0.390 |
| Nuclear Engineering | 1,941 | 0.004 | 0.118 |
| Geosciences | 1,776 | 0.008 | 0.312 |
| Metallurgical Engineering | 1,771 | 0.006 | 0.162 |
| Cognitive Science and Biopsychology | 1,663 | 0.042 | 0.560 |
| Mathematics and Computer Science | 1,599 | 0.009 | 0.333 |
| Nuclear, Industrial Radiology, and Biological Technologies | 1,563 | 0.005 | 0.563 |
| Actuarial Science | 1,498 | 0.007 | 0.391 |
| Mining and Mineral Engineering | 1,452 | 0.002 | 0.106 |
| Physical Sciences | 1,322 | 0.002 | 0.294 |
| Pharmacology | 1,216 | 0.033 | 0.577 |
| Astronomy and Astrophysics | 1,091 | 0.013 | 0.308 |
| Soil Science | 932 | 0.006 | 0.276 |
| Geological and Geophysical Engineering | 856 | 0.003 | 0.255 |
| Military Technologies | 446 | 0.003 | 0.108 |

Notes: *Degree holders* includes all individuals (of any marital status and relation to the household head, age 18-65, men and women, sex not imputed) with a Bachelor’s degree in a certain STEM degree. *Share of coupled men in same-sex couple* is the share of men in a same-sex couple over all coupled men in same-sex or different-sex couples in each field. *Share of individuals that is female* is the share of women (of any marital status and relation to the household head, age 18-65, sex not imputed) over all individuals in each field. Weighted shares using person weights. Only STEM fields reported. Source: ACS 2009-2018.
