# Supplementary material for: Turing’s children: Representation of sexual minorities in STEM
Source: PLoS One. 2020 Nov 18;15(11):e0241596. doi: 10.1371/journal.pone.0241596 (PMC7673532; doi:10.1371/journal.pone.0241596)
Supplement: S13 Table — (DOCX) [file pone.0241596.s020.docx]

**S13 Table. Figure 1 (STEM occupations) in tabular form.**

|  | #Workers | Share of coupled men in same-sex couple | Share of individuals  that is female |
| --- | --- | --- | --- |
| Computer and IT Scientists, Analysts, Specialists, Web Developers | 206,322 | 0.015 | 0.292 |
| Software Developers, Applications, and Systems Software | 112,915 | 0.012 | 0.216 |
| Computer and Information Systems Managers | 59,892 | 0.015 | 0.299 |
| Miscellaneous Engineers, including Nuclear Engineers | 52,779 | 0.006 | 0.138 |
| Computer programmers | 48,110 | 0.011 | 0.233 |
| Engineering Technicians, Except Drafters | 46,235 | 0.005 | 0.198 |
| Civil Engineers | 33,618 | 0.005 | 0.144 |
| Miscellaneous Life, Physical, and Social Science Technicians | 25,798 | 0.014 | 0.477 |
| Mechanical Engineers | 24,887 | 0.003 | 0.085 |
| Network and Computer Systems Administrators | 23,527 | 0.011 | 0.200 |
| Physical scientists, all other | 23,482 | 0.015 | 0.425 |
| Electrical and Electronics Engineers | 22,293 | 0.004 | 0.098 |
| Industrial Engineers, including Health and Safety | 20,683 | 0.006 | 0.212 |
| Architects, Except Naval | 19,504 | 0.027 | 0.291 |
| Drafters | 19,018 | 0.008 | 0.207 |
| Architectural and engineering managers | 16,822 | 0.006 | 0.096 |
| Operations Research Analysts | 15,015 | 0.021 | 0.497 |
| Medical Scientists, and Life Scientists, All Others | 14,415 | 0.023 | 0.536 |
| Aerospace Engineers | 14,255 | 0.004 | 0.132 |
| Database Administrators | 12,298 | 0.015 | 0.391 |
| Biological Scientists | 8,976 | 0.016 | 0.489 |
| Chemists and Materials Scientists | 8,921 | 0.010 | 0.402 |
| Environmental Scientists and Geoscientists | 8,259 | 0.006 | 0.330 |
| Surveying and Mapping Technicians | 7,951 | 0.003 | 0.105 |
| Chemical Technicians | 7,928 | 0.007 | 0.339 |
| Miscellaneous mathematical science occupations | 6,663 | 0.023 | 0.476 |
| Chemical Engineers | 6,268 | 0.004 | 0.175 |
| Computer Hardware Engineers | 5,658 | 0.007 | 0.162 |
| Agricultural and Food Science Technicians | 4,202 | 0.011 | 0.423 |
| Surveyors, Cartographers, and Photogrammetrists | 4,190 | 0.010 | 0.219 |
| Materials Engineers | 3,902 | 0.002 | 0.133 |
| Petroleum, mining, and geological engineers | 3,725 | 0.004 | 0.132 |
| Sales Engineers | 3,507 | 0.004 | 0.083 |
| Environmental Engineers | 3,285 | 0.008 | 0.267 |
| Agricultural and Food Scientists | 3,237 | 0.007 | 0.323 |
| Conservation Scientists and Foresters | 3,034 | 0.005 | 0.217 |
| Actuaries | 2,876 | 0.013 | 0.360 |
| Biological Technicians | 2,799 | 0.020 | 0.466 |
| Natural sciences managers | 2,414 | 0.034 | 0.504 |
| Environmental science and geoscience technicians, and nuclear technicians | 2,242 | 0.008 | 0.246 |
| Biomedical and agricultural engineers | 1,616 | 0.001 | 0.161 |
| Marine engineers and naval architects | 1,496 | 0.007 | 0.108 |
| Atmospheric and space scientists | 1,170 | 0.015 | 0.215 |

Notes: *#Workers* includes all individuals (of any marital status and relation to the household head, age 18-65, men and women, sex not imputed) in a certain STEM occupation. *Share of coupled men in same-sex couple* is the share of men in a same-sex couple over all coupled men in same-sex or different-sex couples in each occupation. Share of individuals that is female is the share of women (of any marital status and relation to the household head, age 18-65, sex not imputed) over all individuals in each occupation. Weighted shares using person weights. Only STEM occupations reported. Source: ACS 2009-2018.
