# Supplementary material for: Turing’s children: Representation of sexual minorities in STEM
Source: PLoS One. 2020 Nov 18;15(11):e0241596. doi: 10.1371/journal.pone.0241596 (PMC7673532; doi:10.1371/journal.pone.0241596)
Supplement: Attachment — Submitted filename: 3_ResponsesToReferees_15Oct2020_final.docx [file pone.0241596.s021.docx]

**Response to Comments from Reviewer 1**

Thank you for the thoughtful review of our paper. We have responded to each of your comments, and we describe these in detail below. Before doing so, however, we begin with an introductory response to your comments. Specifically, we want to highlight that we interpret your Comment 2 as suggesting that in your view all of our baseline models should include the detailed fertility controls you suggest (“any children in the household” and “any young children in the household”). Our original submission had fertility controls as a robustness exercise, but we are happy to oblige the reviewer by including them in our baseline specification. Please note, then, that unless otherwise noted, all of our robustness analyses also include these detailed fertility controls. As we show below in Response Table 1 (which is the original Table 3 plus the detailed fertility controls), controlling for the presence of children in the household, including the presence of young children, does not appreciably change the paper’s main findings. The old Table 3 without fertility controls is now included in the Supporting Information as Table S8. [Please note that to confirm to *PLOS ONE*’s style guidelines what we previously called “Supplemental Appendix” is now called “Supporting Information”, and any table or figure preceded by an “S” appears in the Supporting Information document.]

**Response Table 1: STEM degree and STEM occupation gaps for gay men compared to heterosexual men are robust to controlling for demographic characteristics, fertility, and location.**

|  | ACS 2009-2018 | | | |  | NHIS 2013-2018 | |
| --- | --- | --- | --- | --- | --- | --- | --- |
|  | STEM  degree | | STEM  occupation | |  | STEM  occupation | |
|  | Women | Men | Women | Men |  | Women | Men |
|  | (1) | (2) | (3) | (4) |  | (5) | (6) |
| In a same-sex couple | 0.010^***^ | -0.106^***^ | 0.017^***^ | -0.016^***^ |  |  |  |
|  | (0.002) | (0.003) | (0.001) | (0.001) |  |  |  |
| Gay or lesbian |  |  |  |  |  | 0.003 | -0.014^*^ |
|  |  |  |  |  |  | (0.006) | (0.008) |
| Bisexual |  |  |  |  |  | 0.006 | -0.014 |
|  |  |  |  |  |  | (0.007) | (0.015) |
| Something else |  |  |  |  |  | -0.004 | -0.015 |
|  |  |  |  |  |  | (0.014) | (0.020) |
| Dependent variable mean | 0.139 | 0.345 | 0.032 | 0.095 |  | 0.030 | 0.087 |
| R-squared | 0.030 | 0.039 | 0.015 | 0.029 |  | 0.013 | 0.029 |
| Observations | 2,063,090 | 1,850,340 | 4,664,190 | 4,992,047 |  | 69,972 | 61,890 |

Notes: The dependent variable in columns 1-2 is whether an individual received a bachelor’s degree in a STEM field. The dependent variable in columns 3-6 is whether an individual used to work in a STEM occupation. See also Data and Methodology. All variables are defined in detail in the SI. All regressions include controls for demographic characteristics (age, race, ethnicity), **fertility (indicators for children in the household and children under 5 in the household)**, and location (state fixed effects in the ACS, region fixed effects in the NHIS since we do not observe state of residence in the NHIS public-use data). Weighted regressions using person weights. Standard errors in parentheses. Source: ACS 2009-2018 and NHIS 2013-2018. ^*^ *p* < 0.10, ^**^ *p* < 0.05, ^***^ *p* < 0.01

If we have misinterpreted the Reviewer’s comment/suggestion, we are happy to revert back to the original baseline. Importantly, none of the robustness results reported in this response document or in the revision is critically sensitive to the inclusion of the detailed fertility controls (i.e., the qualitative patterns are unchanged if we exclude them).

**Reviewer 1, Comment 1**: *ACS data. To better enable replication of the paper's approach, it would be helpful to have more detail on the version of the ACS data actually used, for example whether it combined annual (1-year) estimates for each year 2009 through 2018, or took advantage of 3- or 5-year estimates which allow incorporation of greater geographic detail. Since the models seem not to include year fixed effects, I'm presuming pooled estimates, but it would be helpful to know for sure. I would also like to see more explicit discussion of the authors' decision to include state fixed effects in the ACS models, as it seems plausible there may be correlation between share of occupations in STEM fields and sociopolitical attractiveness of a region, due to e.g. presence of universities. If more or fewer STEM jobs are generally available in a region that is relatively unattractive for "out" same-sex couples, it seems like that could influence the results, to the extent that labor is mobile across regions. Sensitivity tests excluding state fixed effects, and excluding California in particular from the sample, would be a start.*

**Response to Reviewer 1, Comment 1**: Thank you for this comment. We clarify in the revision that the analysis relies on the ACS combined annual (1-year) estimates for each year 2009 through 2018. We do not use the 3-year or 5-year estimates.

The reviewer is correct that our original models did not include year fixed effects. Their inclusion does not change any of the paper’s main findings, which we show directly in Response Table 2 below (Table S10 in the revision).

**Response Table 2: STEM degree and STEM occupation gaps as in Table 3. Add year fixed-effects.**

|  | ACS 2009-2018 | | | |  | NHIS 2013-2018 | |
| --- | --- | --- | --- | --- | --- | --- | --- |
|  | STEM  degree | | STEM  occupation | |  | STEM  occupation | |
|  | Women | Men | Women | Men |  | Women | Men |
|  | (1) | (2) | (3) | (4) |  | (5) | (6) |
| In a same-sex couple | 0.010^***^ | -0.107^***^ | 0.017^***^ | -0.017^***^ |  |  |  |
|  | (0.002) | (0.003) | (0.001) | (0.001) |  |  |  |
| Gay or lesbian |  |  |  |  |  | 0.003 | -0.014^*^ |
|  |  |  |  |  |  | (0.006) | (0.008) |
| Bisexual |  |  |  |  |  | 0.006 | -0.016 |
|  |  |  |  |  |  | (0.007) | (0.015) |
| Something else |  |  |  |  |  | -0.004 | -0.017 |
|  |  |  |  |  |  | (0.014) | (0.020) |
| Dependent variable mean | 0.139 | 0.345 | 0.032 | 0.095 |  | 0.030 | 0.087 |
| R-squared | 0.031 | 0.039 | 0.016 | 0.030 |  | 0.013 | 0.030 |
| Observations | 2,063,090 | 1,850,340 | 4,664,190 | 4,992,047 |  | 69,972 | 61,890 |

Notes: The dependent variable in columns 1-2 is whether an individual received a bachelor’s degree in a STEM field. The dependent variable in columns 3-6 is whether an individual used to work in a STEM occupation. Compare to Table 3. See also Data and Methodology. All variables are defined in detail in the SI. All regressions include controls for demographic characteristics (age, race, ethnicity), fertility (indicators for children in the household and children under 5 in the household), location (state fixed effects in the ACS, region fixed effects in the NHIS since we do not observe state of residence in the NHIS public-use data), and **year fixed effects**. Weighted regressions using person weights. Standard errors in parentheses. Source: ACS 2009-2018 and NHIS 2013-2018. ^*^ *p* < 0.10, ^**^ *p* < 0.05, ^***^ *p* < 0.01

The comment about labor mobility across regions is an excellent one, and we agree that it is important to demonstrate that our results are robust to various choices about geography. Below in Response Table 3 (Table S9 in the revision) we show the results from Table 3 where we exclude state fixed effects from the ACS analyses and where we exclude region fixed effects from the NHIS analyses. The estimates are similar to those we reported in the original submission, though the coefficient on the “gay” indicator is no longer statistically significant (p-value=0.102).

**Response Table 3: STEM degree and STEM occupation gaps as in Table 3. No location controls.**

|  | ACS 2009-2018 | | | |  | NHIS 2013-2018 | |
| --- | --- | --- | --- | --- | --- | --- | --- |
|  | STEM  degree | | STEM  occupation | |  | STEM  occupation | |
|  | Women | Men | Women | Men |  | Women | Men |
|  | (1) | (2) | (3) | (4) |  | (5) | (6) |
| In a same-sex couple | 0.013^***^ | -0.110^***^ | 0.018^***^ | -0.013^***^ |  |  |  |
|  | (0.002) | (0.003) | (0.001) | (0.001) |  |  |  |
| Gay or lesbian |  |  |  |  |  | 0.003 | -0.014 |
|  |  |  |  |  |  | (0.006) | (0.008) |
| Bisexual |  |  |  |  |  | 0.006 | -0.013 |
|  |  |  |  |  |  | (0.007) | (0.015) |
| Something else |  |  |  |  |  | -0.004 | -0.014 |
|  |  |  |  |  |  | (0.015) | (0.020) |
| Dependent variable mean | 0.139 | 0.345 | 0.032 | 0.095 |  | 0.030 | 0.087 |
| R-squared | 0.028 | 0.035 | 0.013 | 0.024 |  | 0.013 | 0.029 |
| Observations | 2,063,090 | 1,850,340 | 4,664,190 | 4,992,047 |  | 69,972 | 61,890 |

Notes: The dependent variable in columns 1-2 is whether an individual received a bachelor’s degree in a STEM field. The dependent variable in columns 3-6 is whether an individual used to work in a STEM occupation. Compare to Table 3. See also Data and Methodology. All variables are defined in detail in the SI. All regressions include controls for demographic characteristics (age, race, ethnicity), and fertility (indicators for children in the household and children under 5 in the household). Weighted regressions using person weights. Standard errors in parentheses. Source: ACS 2009-2018 and NHIS 2013-2018. ^*^ *p* < 0.10, ^**^ *p* < 0.05, ^***^ *p* < 0.01

We also agree that it is useful to examine sensitivity to excluding California in the ACS analysis. We do so below in Response Table 4. This additional restriction does not change the main findings. Note that we cannot exclude California from the NHIS analysis because the public use NHIS files do not identify state of residence. We are happy to include this table in the Supporting Information document upon request.

**Response Table 4: STEM degree and STEM occupation gaps as in Table 3. Exclude California.**

|  | ACS 2009-2018  (**excluding California respondents**) | | | |
| --- | --- | --- | --- | --- |
|  | STEM  degree | | STEM  occupation | |
|  | Women | Men | Women | Men |
|  | (1) | (2) | (3) | (4) |
| In a same-sex couple | 0.011^***^ | -0.103^***^ | 0.016^***^ | -0.015^***^ |
|  | (0.002) | (0.003) | (0.001) | (0.001) |
| Dependent variable mean | 0.134 | 0.338 | 0.031 | 0.093 |
| R-squared | 0.029 | 0.034 | 0.014 | 0.025 |
| Observations | 1,822,167 | 1,624,959 | 4,167,614 | 4,428,552 |

Notes: The dependent variable in columns 1-2 is whether an individual received a bachelor’s degree in a STEM field. The dependent variable in columns 3-4 is whether an individual used to work in a STEM occupation. Compare to Table 3. See also Data and Methodology. All variables are defined in detail in the SI. All regressions include controls for demographic characteristics (age, race, ethnicity), fertility (indicators for children in the household and children under 5 in the household), and state fixed effects. Weighted regressions using person weights. Standard errors in parentheses. Source: ACS 2009-2018 (**excluding California respondents**). ^*^ *p* < 0.10, ^**^ *p* < 0.05, ^***^ *p* < 0.01

**Reviewer 1, Comment 2**: *What about the children? One of the arguments for lesbian pay premium (which could be due to working in STEM occupations, which typically require greater human capital investment in higher education) is based on the notion that lesbians are less likely to "opt out" due to childcare. I suggest including a control in the regressions in Table 3 for presence of a child under age 6 as well as any own-child present in the home (as in appendix Table S3), as the apparent lack of impact in the appendix result was, to me, unexpected.*

**Response to Reviewer 1, Comment 2**: Thank you for this comment. We agree that exploring the role of children, including the role of young children, is important. Per the reviewer’s suggestion (and as described in the introductory comment above), we have elevated the models with controls for the presence of children to be the *main* models presented in the paper. [As a minor note, we should mention that, following the IPUMS coding practice (https://usa.ipums.org/usa-action/variables/NCHLT5), we have controlled for the presence of a child under age 5, not under 6.] As shown above in our introductory response, Response Table 1 (Table 3 in the revision) presents this new variant of Table 3 from the original submission and shows that our core findings are qualitatively unaffected by the inclusion of the additional controls for the presence of children in the household, including young children. Unless otherwise specified, we have also included these two fertility controls in all the regression tables in the Supporting Information.

**Reviewer 1, Comment 3a**: *Retention of STEM grads in STEM occupations. For the subset of respondents who do earn STEM degrees, are LGBTQ respondents more or less likely to remain in STEM occupations? Women with bachelor's degrees in STEM fields are less likely than men to persist in STEM occupations, overall, though some of that may be partly driven by an artifact of categorization: health/medical occupations aren't considered STEM, whereas biology majors (popular among premeds) *are* considered STEM. One argument given for women's lower retention is unfriendly work environments as noted in the paper's Discussion section. It would be interesting to know whether LGBTQ graduates seem to be similarly pushed out (again controlling for childrearing).*

**Response to Reviewer 1, Comment 3a**: Thank you for this comment. We agree that retention of STEM degree holders in STEM occupations is a very interesting outcome to examine. In response, we have done two things. First, in Response Table 5 (Table S1 in the revision) we have augmented the prior Table S1 by adding a row to the average STEM occupation outcomes that restrict attention to STEM degree holders. It shows that the raw gap in STEM degrees between men in same-sex couples and men in different-sex couples when we restrict attention to individuals with STEM degrees is much larger than the associated gap when we do not impose this sample restriction.

**Response Table 5: STEM degrees and occupations by type of couple. ACS extensions.**

|  | Women | | |  | Men | | |
| --- | --- | --- | --- | --- | --- | --- | --- |
|  | Same-sex | Different-sex | Gap |  | Same-sex | Different-sex | Gap |
| *STEM degrees:* |  |  |  |  |  |  |  |
| As in Table 1 | 0.140 | 0.139 | 0.001 |  | 0.228 | 0.348 | -0.120^***^ |
| With STEM education | 0.144 | 0.143 | 0.001 |  | 0.230 | 0.351 | -0.121^***^ |
| Without biology | 0.091 | 0.093 | -0.002 |  | 0.172 | 0.297 | -0.125^***^ |
| Without food/nutrition | 0.137 | 0.135 | 0.002 |  | 0.226 | 0.347 | -0.121^***^ |
| With secondary field | 0.149 | 0.145 | 0.004 |  | 0.238 | 0.357 | -0.119^***^ |
| With health | 0.223 | 0.259 | -0.036^***^ |  | 0.276 | 0.375 | -0.099^***^ |
| *STEM occupations:* |  |  |  |  |  |  |  |
| As in Table 1 | 0.050 | 0.032 | 0.018^***^ |  | 0.084 | 0.095 | -0.011^***^ |
| Without agriculture/food | 0.049 | 0.032 | 0.017^***^ |  | 0.084 | 0.094 | -0.010^***^ |
| With physicians/surgeons | 0.051 | 0.033 | 0.018^***^ |  | 0.086 | 0.096 | -0.010^***^ |
| With health (all) | 0.177 | 0.179 | -0.002 |  | 0.164 | 0.131 | 0.033^***^ |
| **Only with STEM degrees** | 0.263 | 0.259 | 0.004 |  | 0.317 | 0.406 | -0.089^***^ |
| *Additional variables:* |  |  |  |  |  |  |  |
| In the labor force | 0.842 | 0.706 | 0.136^***^ |  | 0.852 | 0.884 | -0.032^***^ |
| Unemployed | 0.048 | 0.050 | -0.002^**^ |  | 0.049 | 0.044 | 0.005^***^ |
| Observations | 73,000 | 5,572,796 |  |  | 69,641 | 5,237,089 |  |

Notes: Weighed statistics using person weights. See also Data and Methodology, as well as Table 1. *STEM degree with education* counts as STEM also: Mathematics Teacher Education; Science and Computer Teacher Education. *STEM degree without biology* does not count as STEM: Biology; Biochemical Sciences; Botany; Molecular Biology; Ecology; Genetics; Microbiology; Pharmacology; Physiology; Zoology; Neuroscience; Miscellaneous Biology. *STEM degree without food/nutrition* does not count as STEM: Food Science; Nutrition Sciences. *STEM degree with secondary field* indicates whether an individual received a bachelor’s degree with a primary or secondary concentration in STEM. *STEM degree with health* includes as STEM all majors in the “General Medical and Health Services” IPUMS category. *STEM occupation without agriculture/food* does not count as STEM: agriculture and food scientists; agriculture and food science technicians. *STEM occupation with physicians/surgeons* counts as STEM occupations physicians and surgeons. *STEM occupation with health (all)* counts as STEM occupations all healthcare practitioners, healthcare technical occupations, and healthcare support occupations. *STEM occupation only with STEM degree* is the share of respondents with a STEM occupation only considering respondents with a STEM degree. *Unemployment rate* is computed only among the individuals in the labor force. “Observations” refers to the total number of respondents in the relevant sub-group. Source: ACS 2009-2018. ^*^ *p* < 0.10, ^**^ *p* < 0.05, ^***^ *p* < 0.01

Second, in Response Table 6 (Table 4 in the revision) below we show the results from our main regression-adjusted specification (including controls for fertility) where we restrict attention to individuals with STEM degrees. Note that we can only do this exercise in the ACS, since we do not know STEM degree attainment in the NHIS.

The results in Response Table 6 indicate that conditional on having a STEM degree, men in same-sex couples are significantly less likely to be working in a STEM occupation than otherwise similar men in different-sex couples, and this gap is much larger than the associated gap when we do not restrict the sample to STEM degree holders. Overall, these raw and regression-adjusted patterns are consistent with the reviewer’s hypothesis that sexual minorities are disproportionately ‘pushed out’ of/not retained in STEM occupations even when they have the relevant STEM degrees. We now note this interesting fact in the revision and have elevated it to a main Table in the paper.

**Response Table 6: STEM occupation gaps are larger when focusing on individuals with STEM degrees.**

|  | ACS 2009-2018  (**respondents with STEM degrees only**) | |
| --- | --- | --- |
|  | STEM occupation | |
|  | Women | Men |
|  | (1) | (2) |
| In a same-sex couple | 0.028^***^ | -0.093^***^ |
|  | (0.008) | (0.006) |
| Dependent variable mean | 0.259 | 0.405 |
| R-squared | 0.043 | 0.037 |
| Observations | 252,827 | 622,282 |

Notes: The dependent variable is whether an individual used to work in a STEM occupation. Compare to columns 3-4 in Table 3. See also Data and Methodology. All variables are defined in detail in the SI. All regressions include controls for demographic characteristics (age, race, ethnicity), fertility (indicators for children in the household and children under 5 in the household), and state fixed effects. Weighted regressions using person weights. Standard errors in parentheses. Source: ACS 2009-2018 (**respondents with STEM degrees only**). ^*^ *p* < 0.10, ^**^ *p* < 0.05, ^***^ *p* < 0.01

**Reviewer 1, Comment 3b**: *I'd also like to see some sensitivity checks around the definition of STEM occupations, specifically testing health sciences fields (think e.g. radiology technicians). I also think the inclusion of Nutrition (see Fig. 1) is a mistake; Nutrition degrees are sometimes in Education schools (think K-12 Health teachers) and don't necessarily require much lab science to complete.*

**Response to Reviewer 1, Comment 3b**: Thank you for this comment. We agree that exploring this type of robustness is useful. As described in the Supporting Information, we are relying on independent definitions of what constitutes STEM degrees and STEM occupations from the Department of Commerce for our baseline models. This categorization includes nutrition degrees as STEM degrees. In response to this comment, however, we have augmented Table S1 (which we showed above as Response Table 5) to show the robustness of our main findings to alternative decisions about what fields and occupations to include/exclude in the STEM definition.

Broadly speaking, our results are robust to these choices. In particular, the gaps in STEM degrees and STEM occupations do not change substantially when excluding food and nutrition sciences from the definition of STEM degrees, or when excluding agriculture and food scientists and technicians from the definition of STEM occupations.

**Response Table 5: STEM degrees and occupations by type of couple. ACS extensions.**

|  | Women | | |  | Men | | |
| --- | --- | --- | --- | --- | --- | --- | --- |
|  | Same-sex | Different-sex | Gap |  | Same-sex | Different-sex | Gap |
| *STEM degrees:* |  |  |  |  |  |  |  |
| As in Table 1 | 0.140 | 0.139 | 0.001 |  | 0.228 | 0.348 | -0.120^***^ |
| With STEM education | 0.144 | 0.143 | 0.001 |  | 0.230 | 0.351 | -0.121^***^ |
| **Without biology** | 0.091 | 0.093 | -0.002 |  | 0.172 | 0.297 | -0.125^***^ |
| **Without food/nutrition** | 0.137 | 0.135 | 0.002 |  | 0.226 | 0.347 | -0.121^***^ |
| With secondary field | 0.149 | 0.145 | 0.004 |  | 0.238 | 0.357 | -0.119^***^ |
| **With health** | 0.223 | 0.259 | -0.036^***^ |  | 0.276 | 0.375 | -0.099^***^ |
| *STEM occupations:* |  |  |  |  |  |  |  |
| As in Table 1 | 0.050 | 0.032 | 0.018^***^ |  | 0.084 | 0.095 | -0.011^***^ |
| **Without agriculture/food** | 0.049 | 0.032 | 0.017^***^ |  | 0.084 | 0.094 | -0.010^***^ |
| **With physicians/surgeons** | 0.051 | 0.033 | 0.018^***^ |  | 0.086 | 0.096 | -0.010^***^ |
| **With health (all)** | 0.177 | 0.179 | -0.002 |  | 0.164 | 0.131 | 0.033^***^ |
| Only with STEM degrees | 0.263 | 0.259 | 0.004 |  | 0.317 | 0.406 | -0.089^***^ |
| *Additional variables:* |  |  |  |  |  |  |  |
| In the labor force | 0.842 | 0.706 | 0.136^***^ |  | 0.852 | 0.884 | -0.032^***^ |
| Unemployed | 0.048 | 0.050 | -0.002^**^ |  | 0.049 | 0.044 | 0.005^***^ |
| Observations | 73,000 | 5,572,796 |  |  | 69,641 | 5,237,089 |  |

Notes: Weighed statistics using person weights. See also Data and Methodology, as well as Table 1. *STEM degree with education* counts as STEM also: Mathematics Teacher Education; Science and Computer Teacher Education. *STEM degree without biology* does not count as STEM: Biology; Biochemical Sciences; Botany; Molecular Biology; Ecology; Genetics; Microbiology; Pharmacology; Physiology; Zoology; Neuroscience; Miscellaneous Biology. *STEM degree without food/nutrition* does not count as STEM: Food Science; Nutrition Sciences. *STEM degree with secondary field* indicates whether an individual received a bachelor’s degree with a primary or secondary concentration in STEM. *STEM degree with health* includes as STEM all majors in the “General Medical and Health Services” IPUMS category. *STEM occupation without agriculture/food* does not count as STEM: agriculture and food scientists; agriculture and food science technicians. *STEM occupation with physicians/surgeons* counts as STEM occupations physicians and surgeons. *STEM occupation with health (all)* counts as STEM occupations all healthcare practitioners, healthcare technical occupations, and healthcare support occupations. *STEM occupation only with STEM degree* is the share of respondents with a STEM occupation only considering respondents with a STEM degree. *Unemployment rate* is computed only among the individuals in the labor force. “Observations” refers to the total number of respondents in the relevant sub-group. Source: ACS 2009-2018. ^*^ *p* < 0.10, ^**^ *p* < 0.05, ^***^ *p* < 0.01

On the other hand, when we add health degrees to the STEM degree specification, the raw gap between women in same-sex couples and women in different-sex couples indicates that women in same-sex couples are significantly less likely to have STEM degrees than women in different-sex couples, unlike with the original definition. For men, when we add health degrees we continue to find that men in same-sex couples are significantly less likely to have STEM degree than men in different-sex couples, but the magnitude of the gap is smaller than when we use the original definition. (We note that the patterns using the original STEM degree definition are unaffected when we exclude biology, however).

Regarding STEM occupations, we also find that adding health professions as STEM occupations returns different patterns: for women, when we add health occupations as STEM occupations we do not find any raw gap between women in same-sex couples and women in different-sex couples. For men, however, adding health occupations as STEM occupations returns evidence that men in same-sex couples are significantly *more* likely than men in different-sex couples to be working in a STEM occupation. Table S1 also shows, interestingly, that this sensitivity to recoding health professions as STEM occupations is not driven by the specific case of physicians and surgeons: if we keep the original STEM occupations definition but add only physicians and surgeons we continue to find that men in same-sex couples are significantly less likely than men in different-sex couples to be in STEM occupations.

We are exploring these interesting findings about health professions in a separate paper focusing on physicians and other health professionals. For the revision, we have noted the sensitivity to the categorization of health professions.

**Reviewer 1, Comment 4a**: *Table 3 analysis. I understand the point of including the NHIS data was precisely to deal with the identification limitations of the ACS data; nonetheless, why not include results for the binary indicator "in a same-sex couple" for NHIS data with definition as close as possible to that in the ACS data, to see if the results are consistent?*

**Response to Reviewer 1, Comment 4a**: Thank you for this comment. We agree this is a good idea, though the power issue becomes more much severe when we restrict attention to individuals in couples in the NHIS: while our main models use 2,605 sexual minority women and 2,158 sexual minority men we only observe 561 women in same-sex couples and 440 men in same-sex couples (i.e., 20-25 percent of the sample size when we use sexual minority individuals). For completeness, we report below in Response Table 7 the results of this exercise.

Response Table 7 returns point estimates on the “in a same-sex couple” indicator that are similar in magnitude to those on the “lesbian or gay” indicator when we use individual level information on sexual orientation. [We note that the vast majority of individuals in romantic partnerships who identify as bisexual are in different-sex couples as opposed to same-sex couples, as documented in previous studies.] The estimates are not statistically significant, however, due to the power issues noted above. We present these results only in this response document given these precision challenges. We are happy to include this table in the Supporting Information upon request.

**Response Table 7: STEM occupation gaps in the NHIS looking only at individuals in couples, similar to what we do in ACS.**

|  | NHIS 2013-2018  (**sample adults in same-sex and different-sex couples**) | |
| --- | --- | --- |
|  | STEM occupation | |
|  | Women | Men |
|  | (1) | (2) |
| In a same-sex couple | 0.010 | -0.015 |
|  | (0.010) | (0.016) |
| Dependent variable mean | 0.032 | 0.100 |
| R-squared | 0.017 | 0.031 |
| Observations | 34,673 | 31,799 |

Notes: The dependent variable in columns 1-2 is whether an individual used to work in a STEM occupation. See also Data and Methodology. All variables are defined in detail in the SI. All regressions include controls for demographic characteristics (age, race, ethnicity, fertility), fertility (indicators for children in the household and children under 5 in the household), and location (region fixed effects). Weighted regressions using person weights. Standard errors in parentheses. Source: NHIS 2013-2018. ^*^ *p* < 0.10, ^**^ *p* < 0.05, ^***^ *p* < 0.01

**Reviewer 1, Comment 4b**: *In addition, it's not clear to me why the regressions presented in Table 3 are split by gender, particularly given inclusion of state and region fixed effects. If geography fixed effects are meant to account for e.g. demand-side differences in STEM fields or sociopolitical differences across states/regions, it seems reasonable to assume those effects would be similar for LGBTQ men and women. I would suggest running a single model for each outcome X data set, using indicators for gender and sexual orientation and their interactions to identify these differential effects while holding geographic effects constant. Including race/ethnicity X gender interactions would allow you still to control for intersectionality (as the separate regressions by gender implicitly allow) on those dimensions.*

**Response to Reviewer 1, Comment 4b**: Thank you for this comment. There is substantial evidence that the demand-side differences and sociopolitical differences the reviewer notes are *not* similar for LGBTQ men and women. For example, most studies of labor market outcomes related to sexual orientation find that gay men earn significantly less than similarly situated heterosexual men, while lesbian women earn significantly more than similarly situated heterosexual women. Also, the mean of the outcomes under study are very different across gender, with men being much more likely to have STEM degrees and STEM occupations than women in both datasets (as shown in Tables 1-2). Thus, we prefer to estimate separate models by gender.

For completeness, however, we agree that it is useful to see the model with interactions between gender and sexual orientation. We do so below in Response Table 8. The results in Response Table 8 confirm the patterns in our original submission that men in same-sex couples in the ACS are significantly less likely to have STEM degrees or STEM occupations than similarly situated men in different-sex couples and that gay men in the NHIS are significantly less likely to work in STEM occupations than similarly situated heterosexual men. We are happy to include this table in the Supporting Information upon request.

**Response Table 8: STEM degree and STEM occupation gaps as in Table 3. Pool men and women.**

|  | ACS 2009-2018 | | NHIS 2013-2018 |
| --- | --- | --- | --- |
|  | STEM degree | STEM occupation | Stem occupation |
|  | (1) | (2) | (3) |
| In a same-sex couple | -0.112^***^ | -0.015^***^ |  |
|  | (0.003) | (0.001) |  |
| Woman | -0.212^***^ | -0.065^***^ | -0.059^***^ |
|  | (0.001) | (0.000) | (0.002) |
| In a same-sex couple * Woman | 0.127^***^ | 0.033^***^ |  |
|  | (0.003) | (0.002) |  |
| Gay or lesbian |  |  | -0.019^**^ |
|  |  |  | (0.008) |
| Gay or lesbian * Woman |  |  | 0.027^***^ |
|  |  |  | (0.010) |
| Bisexual |  |  | -0.014 |
|  |  |  | (0.015) |
| Bisexual * Woman |  |  | 0.023 |
|  |  |  | (0.017) |
| Something else |  |  | -0.019 |
|  |  |  | (0.019) |
| Something else * Woman |  |  | 0.020 |
|  |  |  | (0.025) |
| Dependent variable mean | 0.238 | 0.065 | 0.058 |
| R-squared | 0.090 | 0.038 | 0.035 |
| Observations | 3,913,430 | 9,656,237 | 131,862 |

Notes: The dependent variable in columns 1-2 is whether an individual received a bachelor’s degree in a STEM field. The dependent variable in columns 3-6 is whether an individual used to work in a STEM occupation. Compare to Table 3. See also Data and Methodology. All variables are defined in detail in the SI. All regressions include controls for demographic characteristics (age, race, ethnicity), fertility (indicators for children in the household and children under 5 in the household), and location (state fixed effects in the ACS, region fixed effects in the NHIS since we do not observe state of residence in the NHIS public-use data). Weighted regressions using person weights. Standard errors in parentheses. Source: ACS 2009-2018 and NHIS 2013-2018. ^*^ *p* < 0.10, ^**^ *p* < 0.05, ^***^ *p* < 0.01

We have not added race/ethnicity * gender interactions, but the Supporting Information to our original submission did report results by gender and race, which as the reviewer notes also addresses intersectionality.

**Reviewer 1, Comment 4c**: *Also in Table 3, related to comment #3 above, it seems like it would be important to control for educational (degree) attainment, given that there are likely differences in degree attainment by sexual orientation, overall. For example, is the difference in STEM bachelor's degrees due to differences in bachelor's degree attainment overall? And if STEM occupations are more likely to require bachelor's degrees in STEM, how much of the difference in STEM occupations is driven by differences in bachelor's degree attainment, overall?*

**Response to Reviewer 1, Comment 4c**: Thank you for this comment. In Tables S3 and S4 of the original submission we did demonstrate that our result that gay men are significantly less likely to be in STEM occupations than similarly situated heterosexual men is robust to additionally controlling for educational attainment as proxied by having a bachelor’s degree. In fact, the differentials associated with a minority sexual orientation get larger once we add this control for any bachelor’s degree. This is because bachelor’s degree attainment is significantly positively associated with having a STEM occupation, and sexual minority men are significantly more likely to attain any bachelor’s degree (even though they are less likely to attain a STEM degree) than heterosexual men. That is, based on observable characteristics and educational attainment, we would have predicted that the STEM occupation gap between heterosexual men and sexual minority men would have been even larger than what would have been predicted in the absence of the raw educational difference.

The reviewer also raises an interesting and good point that it would also be useful to see the results on STEM degree attainment with the additional control for any bachelor’s degree attainment. We report these results below in Response Table 9 (Table S11 in the revision). [Note that in all other tables the dependent variable “STEM degree” is missing for those with no bachelor’s degree, but for the purposes of this specific exercise those with no bachelor’s degree are assigned a value of zero for STEM degree.]

The results in Response Table 9 indicate that our finding that men in same-sex couples are significantly less likely than men in different-sex couples to have STEM degrees is also robust to controlling for the presence of any BA. We note this in the revision.

**Response Table 9: STEM degree and STEM occupation gaps as in Table 3. Add higher education.**

|  | ACS 2009-2018 | | | |  | NHIS 2013-2018 | |
| --- | --- | --- | --- | --- | --- | --- | --- |
|  | STEM  degree | | STEM  occupation | |  | STEM  occupation | |
|  | Women | Men | Women | Men |  | Women | Men |
|  | (1) | (2) | (3) | (4) |  | (5) | (6) |
| In a same-sex couple | 0.002^**^ | -0.054^***^ | 0.014^***^ | -0.034^***^ |  |  |  |
|  | (0.001) | (0.001) | (0.001) | (0.001) |  |  |  |
| Gay or lesbian |  |  |  |  |  | -0.000 | -0.040^***^ |
|  |  |  |  |  |  | (0.006) | (0.008) |
| Bisexual |  |  |  |  |  | 0.008 | -0.013 |
|  |  |  |  |  |  | (0.007) | (0.015) |
| Something else |  |  |  |  |  | -0.001 | -0.011 |
|  |  |  |  |  |  | (0.014) | (0.019) |
| Dependent variable mean | 0.050 | 0.118 | 0.032 | 0.095 |  | 0.030 | 0.087 |
| R-squared | 0.108 | 0.274 | 0.028 | 0.071 |  | 0.029 | 0.087 |
| Observations | 5,645,796 | 5,306,730 | 4,664,190 | 4,992,047 |  | 69,834 | 61,711 |

Notes: The dependent variable in columns 1-2 is whether an individual received a bachelor’s degree in a STEM field. In contrast with the main analysis, this dependent variable has been set to zero instead of missing for individuals without a bachelor’s degree. The dependent variable in columns 3-6 is whether an individual used to work in a STEM occupation. Compare to Table 3. See also Data and Methodology. All variables are defined in detail in the SI. All regressions include controls for demographic characteristics (age, race, ethnicity), fertility (indicators for children in the household and children under 5 in the household), location (state fixed effects in the ACS, region fixed effects in the NHIS since we do not observe state of residence in the NHIS public-use data), and **an indicator equal to one if an individual’s highest degree completed was a bachelor’s degree or higher**. Weighted regressions using person weights. Standard errors in parentheses. Source: ACS 2009-2018 and NHIS 2013-2018. ^*^ *p* < 0.10, ^**^ *p* < 0.05, ^***^ *p* < 0.01

**Reviewer 1, Comment 5**: *More visuals? One powerful aspect of this journal is the opportunity to provide (more) color Figures to tell the story visually. I like the bottom panel of Figure 1, though I wonder if an exponential might be more appropriate than the linear fit suggested. I'm not sure if you'd have large enough N to exploit the geographic information this way, but something like a heatmap showing which states have larger discrepancies in LGBTQ STEM workforce participation could be interesting. I'd encourage the authors at minimum to consider adding graphical depictions of the descriptive results presented in tables 1 & 2.*

**Response to Reviewer 1, Comment 5**: Thank you for these comments. Regarding the bottom panel of Figure 1 (which is now Figure 3 in the revision) we experimented with both exponential and linear fits and determined that the latter was more appropriate, though of course the punchline of the figure is unaffected by this choice.

We agree that heatmap showing which states have larger discrepancies between men in same-sex couples and men in different-sex couples with respect to STEM degrees and STEM occupations would be interesting. We present this below in Response Figure 1, and we have added it to the Supporting Information as Figure S1. In the figure darker shading indicates greater representation of men in same-sex couples in the STEM degree/occupation relative to men in different-sex couples (weighted using the ACS person weights). Note that the sample size of individuals in same-sex couples is very small in some states, and in some states the gap is negative (i.e., men in same-sex couples are more likely than men in different-sex couples to have a STEM degree/occupation).

**Response Figure 1: STEM degree and STEM occupation gaps between men in same-sex couples and men in different-sex couples, by state (ACS 2009-2018).**

**Panel A: STEM degrees.**

**Panel B: STEM occupations.**

Notes: This map shows the gaps in STEM degrees and STEM occupations between men in same-sex couples and men in different-sex couples in each state. Darker colors indicate that the gaps between the share of men in same-sex couples and the share of men in different-sex couples working or with a STEM degree is smaller (or even positive). Weighed shares using person weights. See also Data and Methodology. Source: ACS 2009-2018.

Finally, following the Reviewer’s suggestion, we have also added to the revision figures that show the results from Tables 1 and 2 in a visual format. We reproduce these below as Response Figures 2 and 3 (Figures 1 and 2 in the revision).

**Response Figure 2: Gaps in STEM degrees and STEM occupations between individuals in same-sex couples and different-sex couples (ACS 2009-2018).**

**Panel A: STEM degree.**

**Panel B: STEM occupation.**

Notes: The number above each bar is the gap between the share of male/female graduates/workers in same-sex couples vs. in different-sex couples in STEM degrees/occupations. Weighed statistics using person weights. See also Data and Methodology, as well as Table 1. All variables are defined in detail in the SI. Source: ACS 2009-2018. ^*^ *p* < 0.10, ^**^ *p* < 0.05, ^***^ *p* < 0.01

**Response Figure 3: Gaps in STEM occupations by sexual orientation (NHIS 2013-2018).**

Notes: The number above each bar is the gap with respect to the share of straight male/female workers in STEM occupations. Weighed statistics using person weights and accounting for survey design. See also Data and Methodology, as well as Table 2. All variables are defined in detail in the SI. Source: NHIS 2013-2018. ^*^ *p* < 0.10, ^**^ *p* < 0.05, ^***^ *p* < 0.01

**Response to Comments from Reviewer 2**

Thank you for the thoughtful review of our paper. We have responded to each of your comments, and we describe these in detail below.

**Reviewer 2, Comment 1**: *The graphs showing the relationship between the fraction of degree holders that are women and the fraction of coupled men in a same-sex couple are fascinating. However, I was left wondering how large each field was. Certainly there is a large amount of variation. I would like to see these graphs made with bubbles of varying size to represent the number of degree holders. Also please note if the linear fit takes into account this weighting. In addition, since this very interesting, releasing the information in tabular format so one can see which field are represented would be pleasing. I feel strongly that these things would improve the interpretability of this paper.*

**Response to Reviewer 2, Comment 1**: Thank you for this comment. We agree that more specificity on this point would improve the paper, and we have done two things in response. First, as requested, we have created a version of the Figure where we change the points to circles indicating the relative number of observations underlying each data point. We reproduce this as Response Figure 4 (Figure S3 in the Supporting Information) below. In addition, that the linear fit in this figure takes account of this weighting (i.e., degrees/occupations with larger underlying sample sizes are given more weight in deriving the linear fit). Note that because there are so many degree fields and occupations we cannot write out the names of each one, and the bubble exacerbates this issue since it would be very difficult to assign text to specific bubbles that overlap other bubbles.

**Response Figure 4: Relationship between share of coupled men in same-sex couples and share women in STEM degrees and STEM occupations (ACS 2009-2018). Weight by field/occupation size.**

**Panel A: STEM degrees.**

**Panel B: STEM occupations.**

Notes: Compare to Figure 3. See also Data and Methodology. The vertical axis measures the share of men in same-sex couples over all coupled women in same-sex or different-sex couples in each field/occupation. Overall, 1.24% of men in a couple are in a same-sex couple. The horizontal axis measures the share of women (of any marital status and relation to the household head, age 18-65, sex not imputed) over all individuals in each field/occupation. Weighted shares using person weights. Only STEM fields/occupations reported. Each circle is proportional to the number of degree holders or workers (of any marital status and relation to the household head, age 18-65, men and women, sex not imputed) in that specific field/occupation. The dashed line plots the linear fit using field/occupation sizes as weights. Source: ACS 2009-2018

Second, also as requested, we added to the Supporting Information two tables showing which degrees fields and occupations are represented in the data in Figures 1 and S3, along with relevant sample sizes and shares. We sort these tables according to the sample size of each degree field/occupation from largest to smallest. We reproduce these as Response Tables 10 and 11 (Tables S12 and S13 in the revision) for degree field and occupation, respectively, below.

**Response Table 10: Figure 1 (STEM degrees) in tabular form.**

|  | #Degree  holders | Share of coupled men in same-sex couple | Share of individuals that is female |
| --- | --- | --- | --- |
| Biology | 192,073 | 0.019 | 0.524 |
| Computer Science | 115,480 | 0.013 | 0.267 |
| Electrical Engineering | 105,725 | 0.007 | 0.140 |
| Mechanical Engineering | 84,035 | 0.005 | 0.107 |
| Mathematics | 72,266 | 0.014 | 0.434 |
| Chemistry | 63,162 | 0.015 | 0.400 |
| General Engineering | 52,817 | 0.006 | 0.168 |
| Civil Engineering | 50,682 | 0.005 | 0.179 |
| Architecture | 41,906 | 0.036 | 0.343 |
| Computer and Information Systems | 37,226 | 0.012 | 0.322 |
| Chemical Engineering | 31,700 | 0.010 | 0.297 |
| Physics | 29,425 | 0.008 | 0.202 |
| Computer Engineering | 28,244 | 0.011 | 0.204 |
| Management Information Systems and Statistics | 22,339 | 0.014 | 0.367 |
| Biochemical Sciences | 21,525 | 0.024 | 0.453 |
| Industrial and Manufacturing Engineering | 20,868 | 0.009 | 0.263 |
| Geology and Earth Science | 18,914 | 0.010 | 0.308 |
| Environmental Science | 18,528 | 0.012 | 0.480 |
| Animal Sciences | 17,871 | 0.010 | 0.556 |
| Transportation Sciences and Technologies | 15,008 | 0.009 | 0.114 |
| Microbiology | 13,916 | 0.021 | 0.555 |
| Zoology | 13,468 | 0.015 | 0.435 |
| Information Sciences | 11,583 | 0.014 | 0.318 |
| Plant Science and Agronomy | 11,517 | 0.015 | 0.324 |
| Electrical Engineering Technology | 11,493 | 0.005 | 0.166 |
| Nutrition Sciences | 11,357 | 0.032 | 0.877 |
| Physiology | 11,198 | 0.017 | 0.544 |
| Aerospace Engineering | 11,171 | 0.007 | 0.113 |
| Industrial Production Technologies | 9,382 | 0.005 | 0.103 |
| Miscellaneous Engineering | 8,690 | 0.006 | 0.214 |
| Miscellaneous Engineering Technologies | 8,479 | 0.003 | 0.205 |
| Operations, Logistics and E-Commerce | 8,370 | 0.011 | 0.326 |
| Molecular Biology | 7,881 | 0.032 | 0.526 |
| Communication Technologies | 7,865 | 0.027 | 0.354 |
| Ecology | 7,761 | 0.018 | 0.556 |
| Miscellaneous Biology | 6,826 | 0.016 | 0.487 |
| Computer Networking and Telecommunications | 6,515 | 0.013 | 0.303 |
| Computer Information Management and Security | 6,297 | 0.014 | 0.264 |
| Biological Engineering | 5,503 | 0.008 | 0.277 |
| Biomedical Engineering | 5,349 | 0.017 | 0.393 |
| Statistics and Decision Science | 4,786 | 0.022 | 0.467 |
| Engineering Technologies | 4,758 | 0.005 | 0.218 |
| Engineering and Industrial Management | 4,724 | 0.004 | 0.198 |
| Applied Mathematics | 4,182 | 0.018 | 0.388 |
| Food Science | 4,154 | 0.017 | 0.614 |
| Materials Engineering and Materials Science | 3,892 | 0.008 | 0.277 |
| Neuroscience | 3,863 | 0.035 | 0.574 |
| Mechanical Engineering Related Technologies | 3,835 | 0.003 | 0.065 |
| Engineering Mechanics, Physics, and Science | 3,471 | 0.010 | 0.149 |
| Computer Programming and Data Processing | 3,412 | 0.017 | 0.338 |
| Environmental Engineering | 3,048 | 0.011 | 0.386 |
| Botany | 2,958 | 0.033 | 0.548 |
| Atmospheric Sciences and Meteorology | 2,612 | 0.021 | 0.263 |
| Petroleum Engineering | 2,487 | 0.009 | 0.152 |
| Materials Science | 2,348 | 0.010 | 0.287 |
| Architectural Engineering | 2,169 | 0.010 | 0.244 |
| *(cont.)* |  |  |  |
| Naval Architecture and Marine Engineering | 2,074 | 0.009 | 0.068 |
| Genetics | 2,002 | 0.019 | 0.557 |
| Oceanography | 1,970 | 0.008 | 0.390 |
| Nuclear Engineering | 1,941 | 0.004 | 0.118 |
| Geosciences | 1,776 | 0.008 | 0.312 |
| Metallurgical Engineering | 1,771 | 0.006 | 0.162 |
| Cognitive Science and Biopsychology | 1,663 | 0.042 | 0.560 |
| Mathematics and Computer Science | 1,599 | 0.009 | 0.333 |
| Nuclear, Industrial Radiology, and Biological Technologies | 1,563 | 0.005 | 0.563 |
| Actuarial Science | 1,498 | 0.007 | 0.391 |
| Mining and Mineral Engineering | 1,452 | 0.002 | 0.106 |
| Physical Sciences | 1,322 | 0.002 | 0.294 |
| Pharmacology | 1,216 | 0.033 | 0.577 |
| Astronomy and Astrophysics | 1,091 | 0.013 | 0.308 |
| Soil Science | 932 | 0.006 | 0.276 |
| Geological and Geophysical Engineering | 856 | 0.003 | 0.255 |
| Military Technologies | 446 | 0.003 | 0.108 |

Notes: *Degree holders* includes all individuals (of any marital status and relation to the household head, age 18-65, men and women, sex not imputed) with a Bachelor’s degree in a certain STEM degree. *Share of coupled men in same-sex couple* is the share of men in a same-sex couple over all coupled men in same-sex or different-sex couples in each field. *Share of individuals that is female* is the share of women (of any marital status and relation to the household head, age 18-65, sex not imputed) over all individuals in each field. Weighted shares using person weights. Only STEM fields reported. Source: ACS 2009-2018.

**Response Table 11: Figure 1 (STEM occupations) in tabular form.**

|  | #Workers | Share of coupled men in same-sex couple | Share of individuals  that is female |
| --- | --- | --- | --- |
| Computer and IT Scientists, Analysts, Specialists, Web Developers | 206,322 | 0.015 | 0.292 |
| Software Developers, Applications, and Systems Software | 112,915 | 0.012 | 0.216 |
| Computer and Information Systems Managers | 59,892 | 0.015 | 0.299 |
| Miscellaneous Engineers, including Nuclear Engineers | 52,779 | 0.006 | 0.138 |
| Computer programmers | 48,110 | 0.011 | 0.233 |
| Engineering Technicians, Except Drafters | 46,235 | 0.005 | 0.198 |
| Civil Engineers | 33,618 | 0.005 | 0.144 |
| Miscellaneous Life, Physical, and Social Science Technicians | 25,798 | 0.014 | 0.477 |
| Mechanical Engineers | 24,887 | 0.003 | 0.085 |
| Network and Computer Systems Administrators | 23,527 | 0.011 | 0.200 |
| Physical scientists, all other | 23,482 | 0.015 | 0.425 |
| Electrical and Electronics Engineers | 22,293 | 0.004 | 0.098 |
| Industrial Engineers, including Health and Safety | 20,683 | 0.006 | 0.212 |
| Architects, Except Naval | 19,504 | 0.027 | 0.291 |
| Drafters | 19,018 | 0.008 | 0.207 |
| Architectural and engineering managers | 16,822 | 0.006 | 0.096 |
| Operations Research Analysts | 15,015 | 0.021 | 0.497 |
| Medical Scientists, and Life Scientists, All Others | 14,415 | 0.023 | 0.536 |
| Aerospace Engineers | 14,255 | 0.004 | 0.132 |
| Database Administrators | 12,298 | 0.015 | 0.391 |
| Biological Scientists | 8,976 | 0.016 | 0.489 |
| Chemists and Materials Scientists | 8,921 | 0.010 | 0.402 |
| Environmental Scientists and Geoscientists | 8,259 | 0.006 | 0.330 |
| Surveying and Mapping Technicians | 7,951 | 0.003 | 0.105 |
| Chemical Technicians | 7,928 | 0.007 | 0.339 |
| Miscellaneous mathematical science occupations | 6,663 | 0.023 | 0.476 |
| Chemical Engineers | 6,268 | 0.004 | 0.175 |
| Computer Hardware Engineers | 5,658 | 0.007 | 0.162 |
| Agricultural and Food Science Technicians | 4,202 | 0.011 | 0.423 |
| Surveyors, Cartographers, and Photogrammetrists | 4,190 | 0.010 | 0.219 |
| Materials Engineers | 3,902 | 0.002 | 0.133 |
| Petroleum, mining, and geological engineers | 3,725 | 0.004 | 0.132 |
| Sales Engineers | 3,507 | 0.004 | 0.083 |
| Environmental Engineers | 3,285 | 0.008 | 0.267 |
| Agricultural and Food Scientists | 3,237 | 0.007 | 0.323 |
| Conservation Scientists and Foresters | 3,034 | 0.005 | 0.217 |
| Actuaries | 2,876 | 0.013 | 0.360 |
| Biological Technicians | 2,799 | 0.020 | 0.466 |
| Natural sciences managers | 2,414 | 0.034 | 0.504 |
| Environmental science and geoscience technicians, and nuclear technicians | 2,242 | 0.008 | 0.246 |
| Biomedical and agricultural engineers | 1,616 | 0.001 | 0.161 |
| Marine engineers and naval architects | 1,496 | 0.007 | 0.108 |
| Atmospheric and space scientists | 1,170 | 0.015 | 0.215 |

Notes: *#Workers* includes all individuals (of any marital status and relation to the household head, age 18-65, men and women, sex not imputed) in a certain STEM occupation. *Share of coupled men in same-sex couple* is the share of men in a same-sex couple over all coupled men in same-sex or different-sex couples in each occupation. Share of individuals that is female is the share of women (of any marital status and relation to the household head, age 18-65, sex not imputed) over all individuals in each occupation. Weighted shares using person weights. Only STEM occupations reported. Source: ACS 2009-2018.

**Reviewer 2, Comment 2**: *Related to the above, I was left wondering if there was a relationship between the fraction of gay men in a field and other measures of diversity. One could imagine that acceptance of feminine people in particular might mean that this field feels more welcoming to gay men, however, that this relationship does not translate to other minoritized groups. Is there a relationship between the fraction of Black men and men in same-sex couples? What about physical disabilities (though I recognize that these may have developed after college)? These answers, sadly, are likely to be relegated to the appendix.*

**Response to Reviewer 2, Comment 2**: Thank you for this comment. We agree investigating other aspects of diversity and their relationship with the fraction of gay men in a field is interesting. We show these relationships below in Response Figures 5 and 6 for race and disability status, respectively (Figures S4 and S5 in the Supporting Information). For race, we used the share identifying as Black or African American; for disability, we used the share with any disability as defined in the IPUMS ACS, including: ambulatory difficulty, independent living difficulty, cognitive difficulty, difficulty taking care of own personal needs, and vision or hearing difficulty.

Neither Response Figure 5 nor Response Figure 6 shows the same strong positive relationship with the fraction of gay men in a field as the share female. For African American race in Response Figure 5 there is a weak positive relationship, while for disability status in Response Figure 6 there is a negative relationship. Thus, the relationship between the fraction of gay men in a field and other minority or underrepresented groups is unique to the share female in the degree/occupation. We note this fact in the revision.

**Response Figure 5: Relationship between share of coupled men in same-sex couples and share black or African American men in STEM degrees and STEM occupations (ACS 2009-2018).**

**Panel A: STEM degrees.**

**Panel B: STEM occupations.**

Notes: The vertical axis measures the share of men in same-sex couples over all coupled men in each field/occupation. Overall, 1.24% of men in a couple are in a same-sex couple. The horizontal axis measures the share of black or African American men (of any marital status and relation to the household head, age 18-65, sex not imputed) over all men in each field/occupation. Overall, 12.34% of men (of any marital status and relation to the household head, age 18-65, sex not imputed) are black or African American. Weighed shares using person weights. See also Data and Methodology. Only STEM fields/occupations reported. The dashed line plots the linear fit. Source: ACS 2009-2018.

**Response Figure 6: Relationship between share of coupled men in same-sex couples and share of individuals with any disability in STEM degrees and STEM occupations (ACS 2009-2018).**

**Panel A: STEM degrees.**

**Panel B: STEM occupations.**

Notes: The vertical axis measures the share of men in same-sex couples over all coupled men in each field/occupation. Overall, 1.24% of men in a couple are in a same-sex couple. The horizontal axis measures the share of individuals (of any marital status and relation to the household head, age 18-65, men and women, sex not imputed) with any disability over all men in each field/occupation. Overall, 10.7% of individuals (of any marital status and relation to the household head, age 18-65, men and women, sex not imputed) have a disability coded in the IPUMS ACS as ambulatory difficulty, independent living difficulty, cognitive difficulty, self-care difficulty, vision or hearing difficulty. Weighed shares using person weights. See also Data and Methodology. Only STEM fields/occupations reported. The dashed line plots the linear fit. Source: ACS 2009-2018.

**Reviewer 2, Comment 3**: *I was intrigued that a positive coefficient appeared sometimes for same-sex partnered women. I understand the desire to not make too much of a result that only appears sometimes in the ACS and not in the NHIS. However, I would at least like to know which controls lead to this coefficient appearing. Particularly as there seems like there is a (very weak) negative relationship between the fraction of women in same-sex couples in a field and the fraction of women in general.*

**Response to Reviewer 2, Comment 3**: Thank you for this comment. We agree it is interesting to examine which controls are driving the small positive associations between being in a same-sex couple and having a STEM degree or a STEM occupation for women. We explore this in more detail below in Response Tables 12, 13, and 14 (Tables S4, S5, and S6 in the revision). Response Table 12 excludes all controls, Response Table 13 only controls for age, and Response Table 14 only controls for race/ethnicity. Since the raw gap in Table 1 and the adjusted gap in Table 3 are the same for STEM occupation for women in the ACS, we focus particular attention on the STEM degree outcome for which the raw gap in Table 1 is zero but the adjusted gap in Table 3 suggests that women in same-sex couples are 1 percentage points more likely than otherwise similar women in different-sex couples to have a STEM degree.

The patterns in Response Tables 12-14 are quite interesting. Response Table 13 indicates that controlling for age alone does not lead to the finding that women in same-sex couples are significantly more likely to have STEM degrees. In contrast, Response Table 14 indicates that controlling for race/ethnicity alone is sufficient to generate the pattern that women in same-sex couples are significantly more likely to have STEM degrees than same-race women in different-sex couples. This is consistent with the patterns presented in Table S2 (already included in the original manuscript as Table S1 Panel B) where we showed raw differences in outcomes across race groups: for women, within-race gaps returned evidence that white women and African American women (who comprise 93 percent of the total sample) in same-sex couples were more likely to have STEM degrees than same-race women in different-sex couples.

**Response Table 12: STEM degree and STEM occupation gaps as in Table 3. No controls.**

|  | ACS 2009-2018 | | | |  | NHIS 2013-2018 | |
| --- | --- | --- | --- | --- | --- | --- | --- |
|  | STEM  degree | | STEM  occupation | |  | STEM  occupation | |
|  | Women | Men | Women | Men |  | Women | Men |
|  | (1) | (2) | (3) | (4) |  | (5) | (6) |
| In a same-sex couple | 0.001 | -0.120^***^ | 0.018^***^ | -0.010^***^ |  |  |  |
|  | (0.002) | (0.003) | (0.001) | (0.001) |  |  |  |
| Gay or lesbian |  |  |  |  |  | 0.004 | -0.023^***^ |
|  |  |  |  |  |  | (0.006) | (0.008) |
| Bisexual |  |  |  |  |  | 0.007 | -0.011 |
|  |  |  |  |  |  | (0.007) | (0.016) |
| Something else |  |  |  |  |  | -0.003 | -0.021 |
|  |  |  |  |  |  | (0.015) | (0.019) |
| Dependent variable mean | 0.139 | 0.345 | 0.032 | 0.095 |  | 0.030 | 0.087 |
| R-squared | 0.000 | 0.001 | 0.000 | 0.000 |  | 0.000 | 0.000 |
| Observations | 2,063,090 | 1,850,340 | 4,664,190 | 4,992,047 |  | 69,972 | 61,890 |

Notes: The dependent variable in columns 1-2 is whether an individual received a bachelor’s degree in a STEM field. The dependent variable in columns 3-6 is whether an individual used to work in a STEM occupation. Compare to Table 3. See also Data and Methodology. All variables are defined in detail in the SI. Weighted regressions using person weights. Standard errors in parentheses. Source: ACS 2009-2018 and NHIS 2013-2018. ^*^ *p* < 0.10, ^**^ *p* < 0.05, ^***^ *p* < 0.01

**Response Table 13: STEM degree and STEM occupation gaps as in Table 3. Control for age.**

|  | ACS 2009-2018 | | | |  | NHIS 2013-2018 | |
| --- | --- | --- | --- | --- | --- | --- | --- |
|  | STEM  degree | | STEM  occupation | |  | STEM  occupation | |
|  | Women | Men | Women | Men |  | Women | Men |
|  | (1) | (2) | (3) | (4) |  | (5) | (6) |
| In a same-sex couple | 0.003 | -0.121^***^ | 0.017^***^ | -0.011^***^ |  |  |  |
|  | (0.002) | (0.003) | (0.001) | (0.001) |  |  |  |
| Gay or lesbian |  |  |  |  |  | 0.004 | -0.023^***^ |
|  |  |  |  |  |  | (0.006) | (0.008) |
| Bisexual |  |  |  |  |  | 0.007 | -0.011 |
|  |  |  |  |  |  | (0.007) | (0.016) |
| Something else |  |  |  |  |  | -0.003 | -0.021 |
|  |  |  |  |  |  | (0.015) | (0.019) |
| Dependent variable mean | 0.139 | 0.345 | 0.032 | 0.095 |  | 0.030 | 0.087 |
| R-squared | 0.004 | 0.002 | 0.000 | 0.000 |  | 0.000 | 0.000 |
| Observations | 2,063,090 | 1,850,340 | 4,664,190 | 4,992,047 |  | 69,972 | 61,890 |

Notes: The dependent variable in columns 1-2 is whether an individual received a bachelor’s degree in a STEM field. The dependent variable in columns 3-6 is whether an individual used to work in a STEM occupation. Compare to Table 3. See also Data and Methodology. All variables are defined in detail in the SI. All regressions include controls for demographic characteristics (**age only**). Weighted regressions using person weights. Standard errors in parentheses. Source: ACS 2009-2018 and NHIS 2013-2018. ^*^ *p* < 0.10, ^**^ *p* < 0.05, ^***^ *p* < 0.01

**Response Table 14: STEM degree and STEM occupation gaps as in Table 3. Control for race/ethnicity.**

|  | ACS 2009-2018 | | | |  | NHIS 2013-2018 | |
| --- | --- | --- | --- | --- | --- | --- | --- |
|  | STEM  degree | | STEM  occupation | |  | STEM  occupation | |
|  | Women | Men | Women | Men |  | Women | Men |
|  | (1) | (2) | (3) | (4) |  | (5) | (6) |
| In a same-sex couple | 0.013^***^ | -0.111^***^ | 0.020^***^ | -0.010^***^ |  |  |  |
|  | (0.002) | (0.003) | (0.001) | (0.001) |  |  |  |
| Gay or lesbian |  |  |  |  |  | 0.006 | -0.018^**^ |
|  |  |  |  |  |  | (0.006) | (0.008) |
| Bisexual |  |  |  |  |  | 0.009 | -0.015 |
|  |  |  |  |  |  | (0.007) | (0.015) |
| Something else |  |  |  |  |  | -0.000 | -0.017 |
|  |  |  |  |  |  | (0.015) | (0.020) |
| Dependent variable mean | 0.139 | 0.345 | 0.032 | 0.095 |  | 0.030 | 0.087 |
| R-squared | 0.025 | 0.035 | 0.012 | 0.024 |  | 0.012 | 0.028 |
| Observations | 2,063,090 | 1,850,340 | 4,664,190 | 4,992,047 |  | 69,972 | 61,890 |

Notes: The dependent variable in columns 1-2 is whether an individual received a bachelor’s degree in a STEM field. The dependent variable in columns 3-6 is whether an individual used to work in a STEM occupation. Compare to Table 3. See also Data and Methodology. All variables are defined in detail in the SI. All regressions include controls for demographic characteristics (**race and ethnicity only**). Weighted regressions using person weights. Standard errors in parentheses. Source: ACS 2009-2018 and NHIS 2013-2018. ^*^ *p* < 0.10, ^**^ *p* < 0.05, ^***^ *p* < 0.01

**Reviewer 2, Comment 4**: *Finally, pure speculation, looking at the chart of fraction of women in a field and the fraction of women in same-sex couples in a field, I wondered about the “academicness” of fields. Something like “Nuclear, Industrial Radiology, and Biological Technologies” is not only a small field, but very vocational. It prepares people to do power plant and infrastructure work, it is not a field that appears at any LACs or that there exist PhD programs in. This may be an extreme outlier but looking at the appendix graphs of women in same-sex couple I wonder about some pattern, as the fields with more women in same-sex couples seem to be in more “academic” fields. I partly wonder about this because of the way gender roles are expressed in the “occupational hierarchy” in that occupations that require less education tend to be more gender-segregated. I don’t know if this holds for people who are choosing more vocational majors, but I suspect it does. I wonder if less vocational fields are more or less likely to be chosen by those in a same sex couple.*

**Response to Reviewer 2, Comment 4**: Thank you for this very interesting comment. We explored this further by plotting the “lesbian share” (i.e., the share of all coupled women that is composed of women in same-sex couples) against the share of all individuals in the degree or occupation that has a PhD, which we think is a reasonable proxy for the “academicness” that the reviewer describes. As noted, this will capture the fact that the more vocational fields/occupations are less likely to have PhD programs. We present this information below in Response Figure 7.

Response Figure 7 shows that there is a weak *negative* relationship between the “academicness” of a field or occupation (as proxied by the share with a PhD) and the representation of women in same-sex couples. We are happy to include this in the Supporting Information upon request.

**Response Figure 7: Relationship between share of coupled women in same-sex couples and share individuals with Ph.D. in STEM degrees and STEM occupations (ACS 2009-2018).**

**Panel A: STEM degrees.**

**Panel B: STEM occupations**

Notes: The vertical axis measures the share of women in same-sex couples over all coupled men in each field/occupation. Overall, 1.27% of women in a couple are in a same-sex couple. The horizontal axis measures the share of individuals with a PhD (of any marital status and relation to the household head, age 18-65, men and women, sex not imputed) over all individuals in each field/occupation. Overall, 1.05% of individuals (of any marital status and relation to the household head, age 18-65, men and women, sex not imputed) have a PhD. Weighed shares using person weights. See also Data and Methodology. Only STEM fields/occupations reported. The dashed line plots the linear fit. Source: ACS 2009-2018.

**Response to Comments from Reviewer 3**

Thank you for the thoughtful review of our paper. We have responded to each of your comments, and we describe these in detail below.

**Reviewer 3, Comment 1**: *It would be good to have a brief discussion of the definition of STEM. The details can stay in the Appendix, but the paper needs a little bit on this.*

**Response to Reviewer 3, Comment 1**: Thank you for this comment. We agree that the manuscript itself needs greater discussion of the definition of STEM. In response, we have moved some of the Supporting Information material into the main text, so the definition of STEM is clearer. Specifically, we list several categories of examples for both STEM degrees and STEM occupations.

**Reviewer 3, Comment 2**: *Also, do a check for typos.*

**Response to Reviewer 3, Comment 2**: We have checked the paper and Supporting Information for typographical errors.
